# Thermochemical Activation of Carbon Steel EAF and FeCr Slags for Chromium and Vanadium Leaching

**DOI:** 10.3390/ma19153213

**Published:** 2026-07-28

**Authors:** Andrea Miškufová, Zita Takáčová, Jana Pirošková, Olívia Melegová, Dagmar Remeteiová, Jaroslav Briančin

**Affiliations:** 1 Faculty of Materials, Metallurgy and Recycling, Institute of Recycling and Environmental Technologies, Technical University of Kosice, Letna 1/9, 042 00 Kosice-Sever, Slovakia; zita.takacova@tuke.sk (Z.T.); jana.piroskova@tuke.sk (J.P.); dagmar.remeteiova@tuke.sk (D.R.); 2 U. S. Steel, s.r.o. Kosice, Vstupny areal U. S. Steel, 044 54 Kosice, Slovakia; olivia.melegova@gmail.com; 3 Institute of Geotechnics, Slovak Academy of Sciences, Watsonova 45, 040 01 Kosice, Slovakia; briancin@saske.sk

**Keywords:** thermochemical activation, EAF slag, FeCr Slag, NaOH, leaching, chromium, vanadium

## Abstract

This study presents a novel, low-temperature thermochemical activation process for the selective extraction of Cr and V from carbon steel EAF (CH1) and FeCr (CH2) slags at temperatures of up to 600 °C. Of the twelve reagents tested, NaOH was identified as the optimal alkaline agent for Cr activation at 500 °C, achieving extraction yields of 61.6% for CH1 (slag-to-reagent ratio of 12:8 g) and 80.6% for CH2 (ratio of 12:16 g). KOH at 400 °C was the most effective reagent for V extraction, yielding 89.4% for CH1 and 54.5% for CH2. Maximum metal concentrations were achieved after only five minutes of leaching at 60 °C. The process exhibits high selectivity; primary matrix components (Fe, Si, Al, Ca, Mg) either do not leach or only leach in negligible amounts. Iron forms insoluble oxides, and calcium converts into stable calcite, while magnesium is bound in the form of hydrotalcite specifically in the CH2 slag leaching residue. The CaCO_3_ content was proven to be a crucial parameter determining the activation efficiency and effective transformation of Fe-Cr-V phases. This procedure enables the recovery of clean Cr and V leachates, while the residual mineral-rich fraction offers potential for various industrial applications in a closed-loop slag recycling process.

## 1. Introduction

Steel is the world’s most used and recycled metal. Global crude steel production reached 1882.6 million tonnes in 2024, with forecasts reaching 2.5 billion tonnes by 2050 [1,2]. While the basic oxygen furnace (BOF) route remains dominant (71.1%), electric arc furnaces (EAFs) account for about 45% of production in the EU-27, with further growth expected as steel scrap recycling increases [3]. This shift toward electric steelmaking is vital for industrial decarbonization but generates a considerable amount of by-products: in 2024, global steel slag production was estimated at 190–290 million tonnes, 26% of which was carbon steel EAF slag [4,5,6]. Although developed countries valorize up to 97% of these materials—primarily in road construction—they face a fundamental challenge: the loss of valuable contained metallic elements [7,8,9]. Given that vanadium is classified as a Critical Raw Material (CRM) in the EU, and chromium and nickel are ranked among high-economic and strategic metals, their recovery is essential for supply security and aligns with the principles of the circular economy by transforming industrial waste into high-value resources [10].

EAF and ferrochromium (FeCr) slags represent valuable secondary sources of metals, including Cr, V, Mo, Mn, and Nb. The treatment of these slags traditionally involves mechanical–physical pretreatment, such as magnetic separation, gravity separation, and integrated combinations of optical and vibrating techniques [11,12,13,14,15]. These processes, involving cooling, crushing, grinding, and detailed separation steps, aim to liberate residual metallic phases—specifically entrapped Fe, Cr, and other alloying elements—from the inert slag matrix [9,16,17,18,19,20,21,22,23,24,25]. This ensures the production of high-grade metallic concentrates while simultaneously upgrading the slag for downstream applications in construction or civil engineering [19,21,22,23,24].

To address the limitations of the mineral phases remaining after physical separation, research has extensively explored pyrometallurgical, hydrometallurgical, and combined processing routes. While pyrometallurgical reduction smelting is a well-established technology [26,27,28], hydrometallurgical methods have garnered significant attention in recent years due to their potential for lower energy consumption and operational flexibility [29,30,31,32,33,34,35]. Despite this widespread research interest, the efficient hydrometallurgical recovery of target metals remains challenging, primarily due to the chemical resistance of stable spinel phases—such as chromite-type structures—which effectively sequester valuable elements like Cr and V. To overcome these structural limitations, the focus of recent studies has shifted toward various combined thermochemical activation and leaching processes [32,33,34,35,36,37,38,39,40,41].

Most of these intensification efforts rely on sodium-based alkaline thermochemical activation, including conventional, microwave (MW)-, and radio-frequency (RF)-assisted methods [36,40,41,42]. Conventional thermochemical activation can achieve nearly 100% Cr extraction; however, it requires energy-intensive conditions (1000 °C, 4 h) [40,41,42]. Conversely, while electromagnetic-assisted techniques (MW and RF) offer notable process intensification—facilitating activation at much lower temperatures (e.g., 280–400 °C)—they often struggle with selectivity [36,40,41,42]. For instance, the MW-based approach has demonstrated a divergence in results: while achieving high Cr extraction for certain slag types, it has resulted in zero vanadium extraction, failing to replicate the efficiency observed in conventional processes [40,41]. Similarly, RF-assisted methods often yield only partial extraction of target metals or prove ineffective without prior thermochemical activation [36,40,41]. As an alternative to these sodium-based routes, calcium-based (lime) thermochemical activation has been investigated to convert Cr into water-soluble species such as calcium chromate; however, this approach requires extended residence times and subsequent complex adsorption steps to recover the metals [43].

Consequently, the inability of these low-temperature intensification methods to consistently recover multiple valuable metals simultaneously, specifically vanadium, represents a critical challenge in industrial slag valorization.

Despite existing research, the efficient recovery of metals from slags remains a significant challenge due to the stable spinel structures that resist conventional leaching. This study presents an innovative approach to processing carbon steel EAF and FeCr slags via low-temperature thermochemical activation (up to 600 °C) using a tailored selection of chemical reagents (e.g., Na_2_O_2_, NaOH-NaCl, NaOH-NaNO_3_, NaOH-Na_2_O_2_, K_2_S_2_O_7_, NaH_2_PO_4_·H_2_O, (NH_4_)_2_SO_4_-KHSO_4_, and KOH). The novelty of this work lies in the high process selectivity achieved at moderate activation temperatures, which facilitates the transformation of chemically resistant spinel or silicate phases containing Cr and V into water-soluble species while minimizing the dissolution of the thermochemically activated carbon steel EAF and FeCr slag matrix (oxides of Fe, Si, Ca, and Mg). Furthermore, this study identifies the decisive influence of the CaCO_3_ content in input slags on thermochemical effectiveness when using NaOH reagent—a mechanism not previously reported for low-temperature activation of both investigated slags (up to 600 °C). Compared to MW heating, conventional heating proves to be a more energy-efficient and scalable solution for industrial-scale processing. Notably, conventional activation enables efficient vanadium extraction at temperatures as low as 500 °C, whereas MW-assisted processes under similar conditions yield negligible results. Building on these findings, the proposed process ensures rapid and selective recovery of Cr and V from the activated slag through aqueous leaching in just 5 min at 60 °C. Furthermore, the study identifies the formation of secondary hydrotalcite-type phases in the FeCr slag leaching residue, providing new insights into the immobilization of matrix components.

## 2. Materials and Methods

### 2.1. Materials

**Figure 1:**
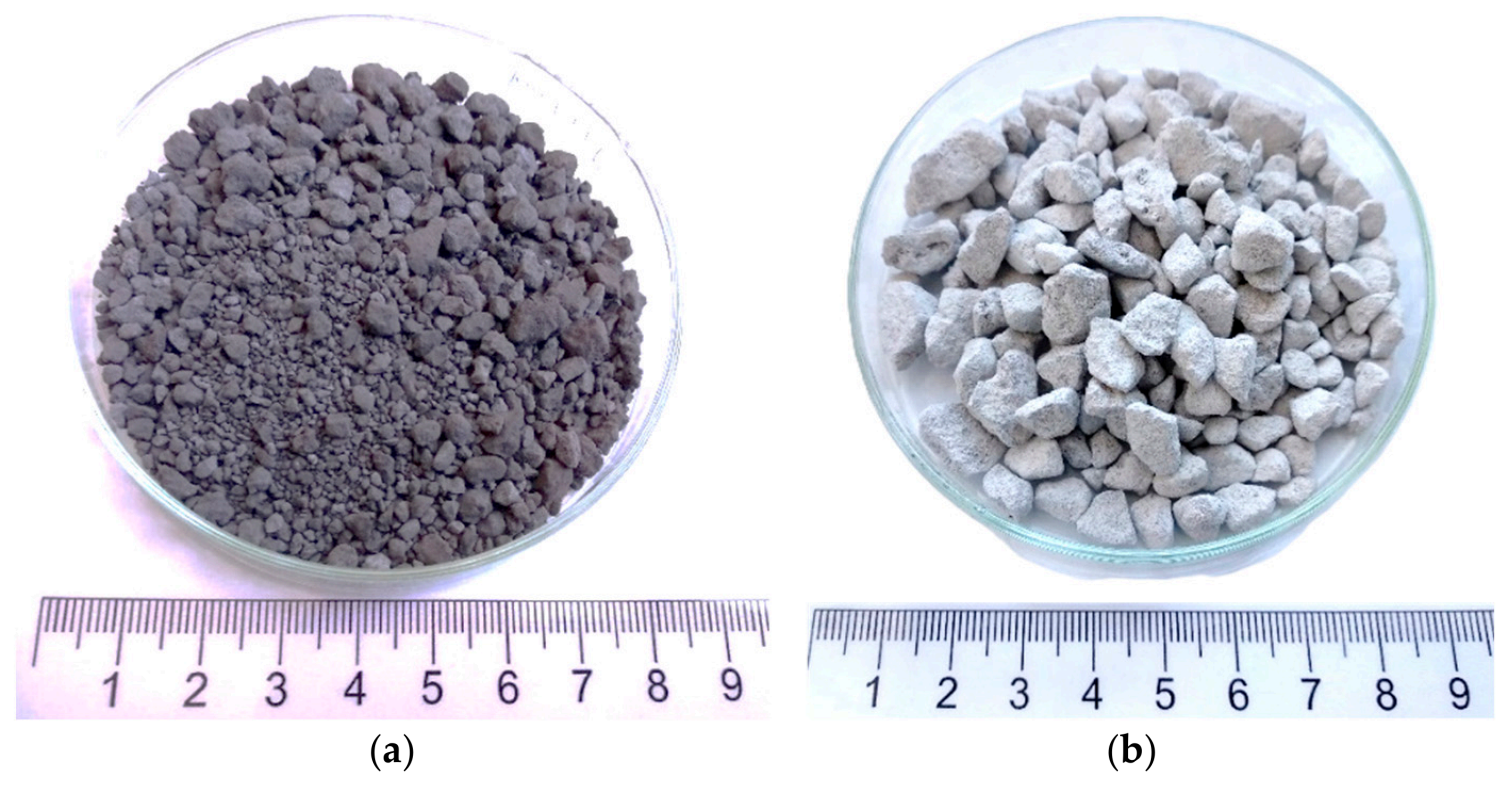
Visual appearance of the investigated slag samples: (**a**) carbon steel EAF slag labelled CH1 and (**b**) FeCr slag labelled CH2.

Two types of slag were utilized in the experimental part of this study (Figure 1): carbon steel EAF slag (labelled CH1) and FeCr slag (labelled CH2). Both samples were provided by partner organizations as part of the international Horizon 2020 Research and Innovation Program under grant agreement No. 730471 (CHROMIC). Following a detailed analysis of their chemical and mineralogical composition, the slag samples underwent mechanical pretreatment in a VM4 vibratory mill (OPS Přerov, Czech Republic). This process was conducted to ensure material homogeneity, reduce particle size, and increase the specific surface area, all of which are essential for maximizing processing efficiency.

### 2.2. Analytical Methods

The detailed morphology of the slag samples was studied using a Dino-Lite Pro AM 413T digital microscope (AnMo Electronics Corp., Taipei, Taiwan) and a Nikon LV150N optical microscope (Nikon Corp., Tokyo, Japan). Material moisture content was determined by loss on drying, using 10 g samples heated at 105 °C for 4 h in a Memmert UN55 oven (Memmert GmbH + Co. KG, Schwabach, Germany).

Chemical composition was analyzed using high-resolution continuum source flame atomic absorption spectrometry (HR-CS FAAS) on a ContrAA 700 spectrometer (Analytik Jena AG, Jena, Germany) operated via ASpect CS software (ver.2.0). For each representative slag type, five samples were collected, and each was analyzed in triplicate to determine average elemental concentrations.

Scanning electron microscopy (SEM) combined with energy-dispersive spectrometry (EDS) was performed using a Carl Zeiss EVO MA15 SEM (Carl Zeiss AG, Oberkochen, Germany) equipped with an Oxford Instruments EDX system (Oxford Instruments plc, Abingdon, UK). To ensure the conductivity of the samples for SEM-EDS analysis, the sample surfaces were coated with gold or carbon. Additionally, the qualitative and semi-quantitative elemental composition and surface distribution of selected thermochemical activation products and leaching residues were analyzed using Laser-Induced Breakdown Spectroscopy (LIBS). These measurements were conducted with a Keyence VHX-X1FE digital optical microscope integrated with an EA-300 LIBS analyzer (Keyence Corp., Osaka, Japan).

Phase composition was determined by X-ray diffraction (XRD) using a Philips X’Pert PRO MRD diffractometer (Co-Kα radiation; Philips, Amsterdam, The Netherlands), with phases identified via X’Pert HighScore Plus software (ver. 3.0a). Magnetic properties were quantified by measuring the coercive force using an ATS 320 coercive meter (Applied Technology & Science, s.r.o., Košice, Slovakia). Material density was determined using an AccuPyc II 1340 automatic helium pycnometer (Micromeritics Instrument Corp., Norcross, GA, USA).

All chemicals used in the experiments were of analytical grade (p.a. quality). During the leaching process, the concentrations of Cr and V were monitored at each sampling interval. Final leachates were also analyzed for primary slag matrix elements, specifically Fe, Si, Ca, and Mg. All liquid samples were analyzed by the HR-CS FAAS method using the contrAA 700 spectrometer (Analytik Jena, Jena, Germany), ensuring consistency with the solid-phase analyses.

### 2.3. Sample Characterization

**Figure 2:**
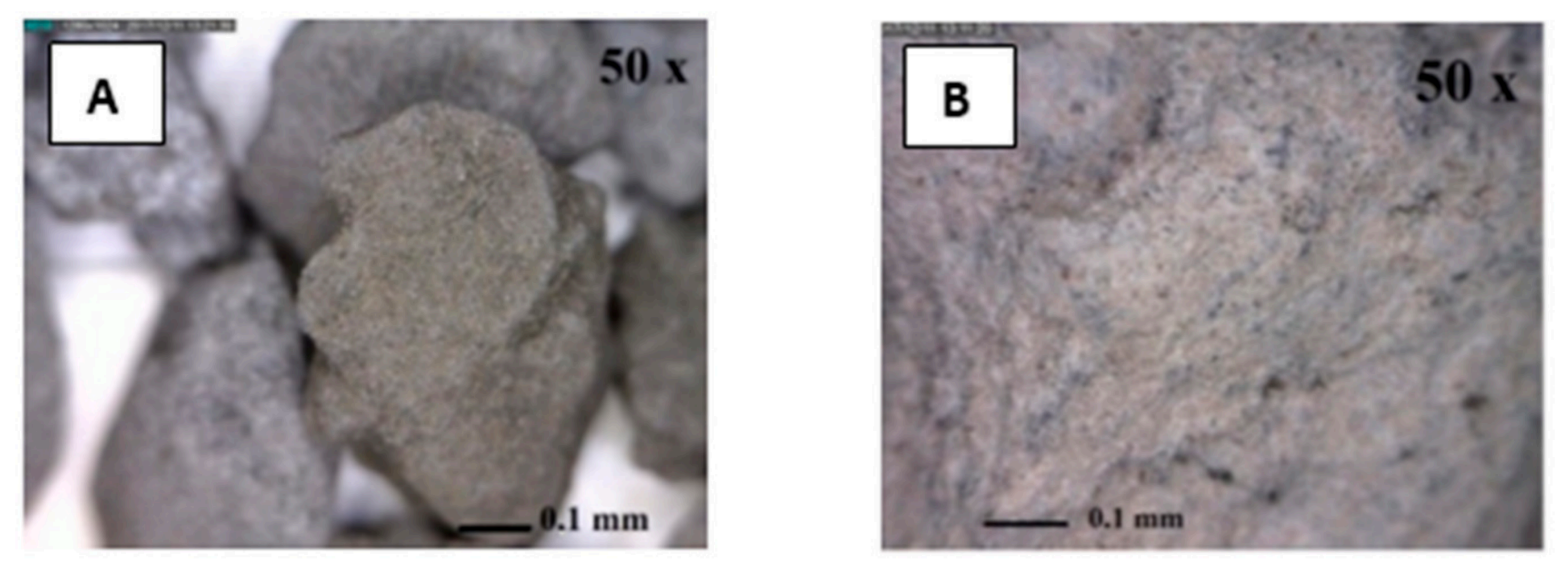
Stereomicroscopic morphology of the original samples at 50× magnification: (**A**) CH1 and (**B**) CH2.

A morphological study at 50× magnification using a stereomicroscope provided insights into the appearance, color, and texture of the slag particles (Figure 2A,B). Sample CH1 (Figure 2A) exhibits a structure characteristic of steelmaking slag with a high Fe content. In contrast, sample CH2 (Figure 2B) displays a distinct character typical of FeCr production slags rich in calcium and magnesium silicates, lacking a high Fe concentration.

**Figure 3:**
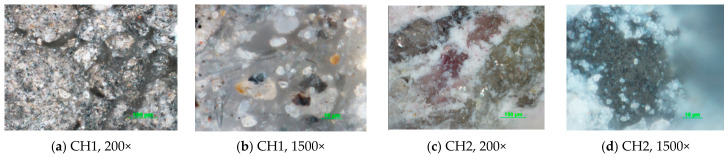
Optical micrographs of the investigated slags: (**a**) CH1 in reflected light (200×), (**b**) CH1 in cross-polarized light (1500×), (**c**) CH2 in reflected light (200×), and (**d**) CH2 in cross-polarized light (1500×).

Optical microscopy analysis (Figure 3) reveals a highly heterogeneous structure in both slags. Sample CH1 (Figure 3a,b), observed in reflected light at 200× magnification and under cross-polarized light at 1500× magnification, shows irregular particles with varying reflectivity, indicating differences in the optical properties of individual grains and confirming the heterogeneous phase composition.

Sample CH2 exhibits an analogously heterogeneous microstructure from the material and granularity composition perspective (Figure 3c,d). Reflected light observations reveal that a metallic inclusion of the FeCr phase is present in the CH2 slag (subsequently confirmed by SEM-EDS analysis).

The concentrations of the major and trace elements in the studied slag samples are summarized in Table 1.

Chemical analysis revealed a slightly higher Cr content in the FeCr production slag (CH2), consistent with its origin and the use of Cr-bearing raw materials during FeCr smelting. Conversely, the carbon steel EAF slag (CH1) exhibited a higher V content (0.072%), reflecting the specific scrap charge composition and the presence of alloying elements during steelmaking.

A considerable discrepancy between the samples analyzed was observed in their Fe content. Sample CH1 (carbon steel EAF slag) contained more Fe compared to the FeCr slag CH2, a result of the differing technological and redox conditions inherent to steel and ferroalloy production. In the EAF steelmaking process, a portion of the Fe oxidizes to FeO and migrates into the slag, facilitating the removal of impurities such as phosphorus. In contrast, the highly reductive conditions during ferroalloy production ensure minimal Fe loss to the slag. This pronounced difference in Fe content is critical for downstream processing; while CH1 represents a potentially valuable secondary source of Fe, CH2 is comparatively depleted of Fe and is more suitable as a fluxing agent or a precursor for construction materials.

Variations were also noted in the concentrations of Mg, Ca, and Si—the primary components of fluxing agents in both processes. Given that the Fe content in CH2 is negligible, the concentrations of these matrix elements (Si, Ca, Mg) are notably higher than in CH1. The distinct Fe levels, overall chemical composition, and physicochemical properties directly govern the reactivity of these slags. These factors are decisive when selecting optimal recycling routes, designing thermal and hydrometallurgical treatments, and defining the potential for final material valorization.

**Figure 4:**
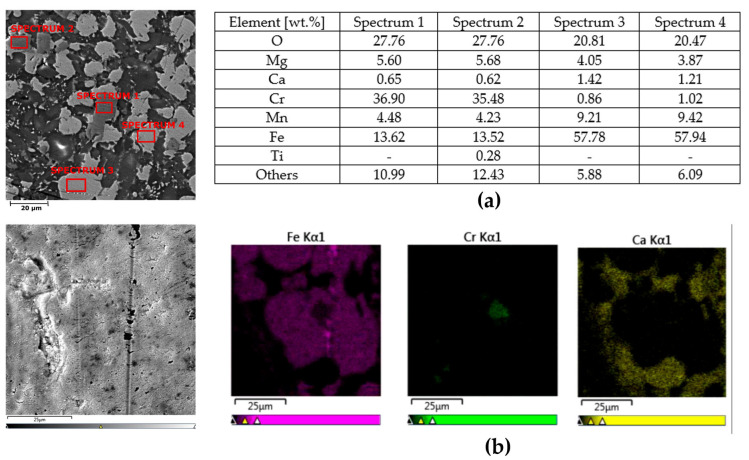
SEM-EDS analysis of the CH1 slag sample: (**a**) point analysis and (**b**) elemental mapping.

SEM-EDS analysis of the CH1 slag sample is presented in Figure 4. EDS point analysis focused on the Cr-rich particles revealed that Cr is associated with Fe, Mg, Mn, and oxygen (spectra 1 and 2 in Figure 4a, dark grey particles). This elemental composition suggests that chromium is present as a complex chromite-type spinel phase, such as (Mn,Fe,Mg)Cr_2_O_4_, in preference to a simple oxide. The light grey particles (Spectrum 3 and 4 in Figure 4a) represent iron oxides that contain a substantially lower amount of chromium and are depleted in manganese. Furthermore, titanium was also detected within the analyzed particles, indicating the presence of titanomagnetite (Spectrum 2 in Figure 4a). Complementary to this, elemental mapping (Figure 4b) shows that Cr-rich particles are localized and isolated, rather than being uniformly dispersed or directly associated with the Fe-Ca matrix. These findings highlight the specific occurrence of Cr within refractory spinel phases, which needs to be taken into account during the thermochemical activation process.

**Figure 5:**
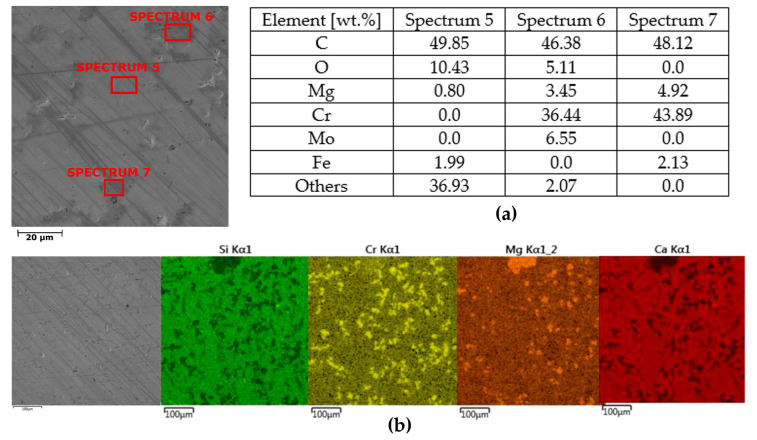
SEM-EDS analysis of the CH2 slag sample: (**a**) point analysis and (**b**) elemental mapping.

For sample CH2, SEM-EDS analysis (Figure 5) confirmed the presence of Ca, Si, Mg, and Cr. Chromium is more evenly distributed within the CH2 matrix compared to in CH1. It is associated with oxygen, likely existing as Cr_2_O_3_ or isolated metallic Cr/FeCr particles. Furthermore, Cr association with Mg suggests the potential presence of MgCr_2_O_4_, as shown in the point SEM-EDS analysis and elemental mapping (Figure 5b). The co-occurrence of Ca, Si, and Mg indicates the presence of various calcium and magnesium silicates. Notably, V and Nb were not detected by SEM-EDS in either CH1 or CH2, likely due to their concentration being below the detection limit of the used analytical method. However, molybdenum was identified in the CH2 sample (Spectrum 6 in Figure 5a).

**Figure 6:**
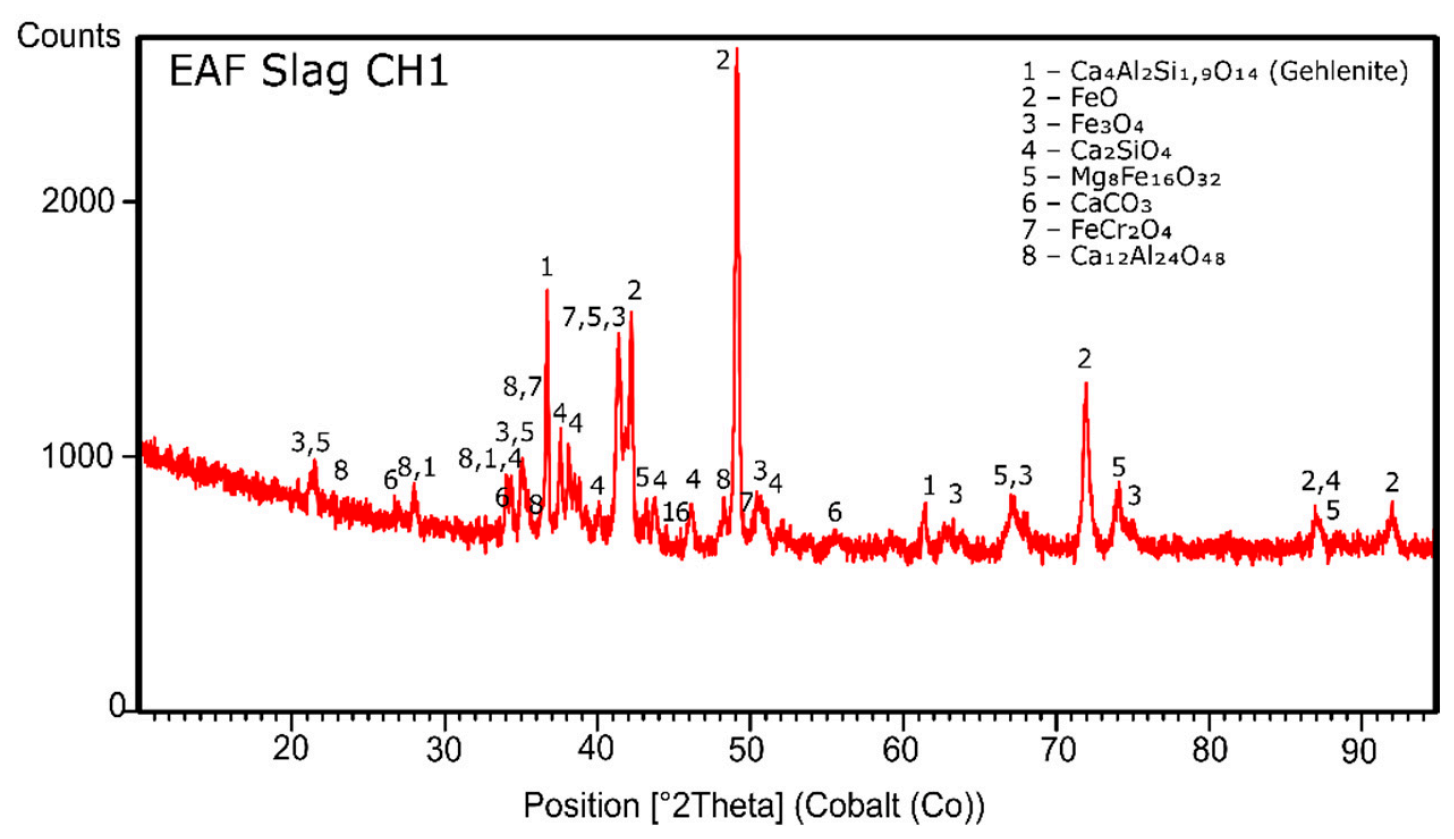
XRD diffraction pattern of the EAF (CH1) slag sample.

**Figure 7:**
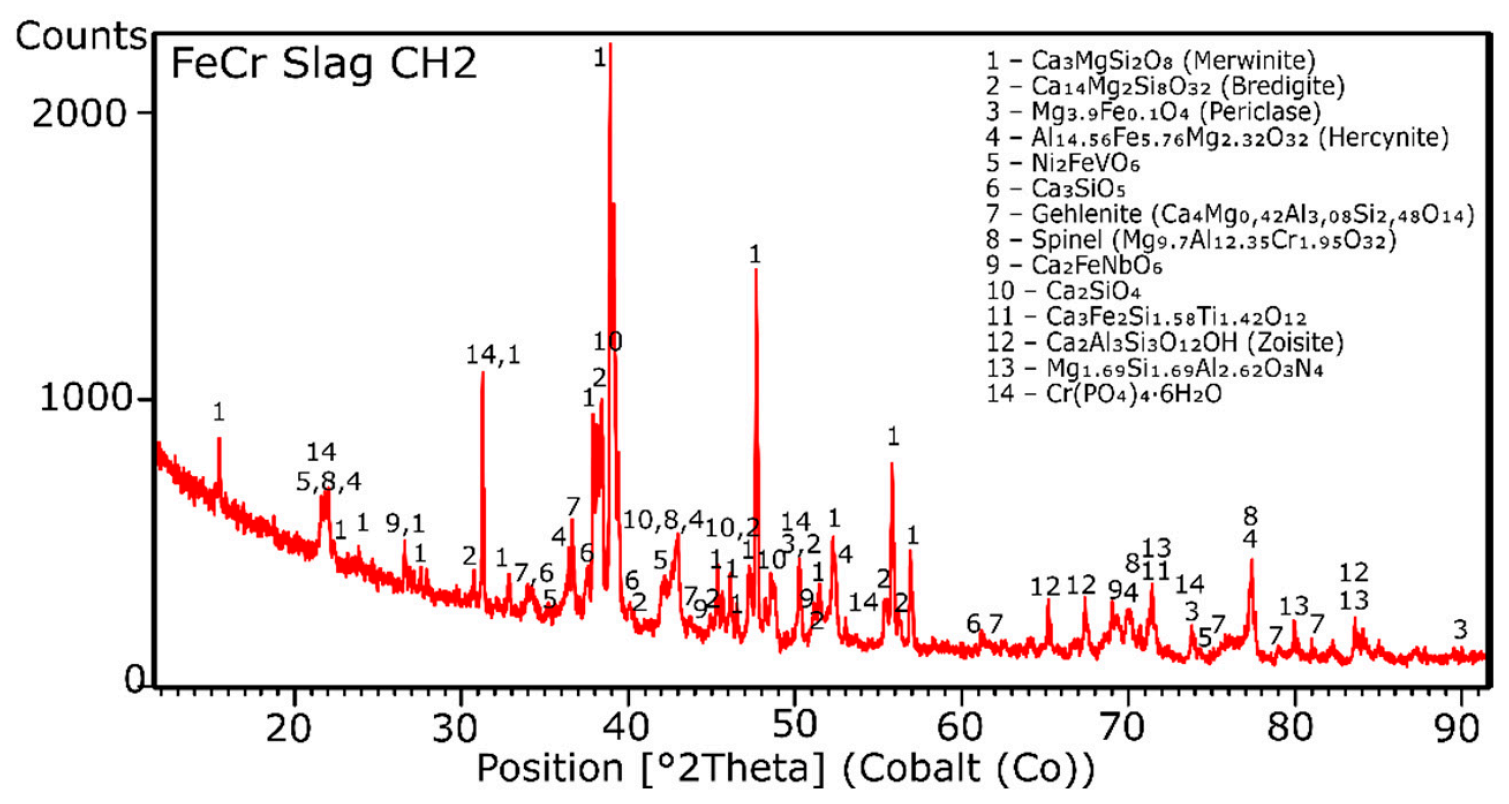
XRD diffraction pattern of the FeCr (CH2) slag sample.

The XRD diffraction patterns for both samples are presented in Figure 6 (CH1) and Figure 7 (CH2).

The diffraction pattern of sample CH1 (Figure 6) reveals that the major phases include wüstite (FeO), gehlenite (calcium aluminosilicate), dicalcium silicate (Ca_2_SiO_4_), magnetite (Fe_3_O_4_), and magnesioferrite (Mg_8_Fe_16_O_32_), which fundamentally correlates with the SEM-EDS findings. Chromium was identified in the form of chromite (FeCr_2_O_4_). Chromite could be associated with Mn and V, a possibility that was also investigated via SEM-EDS. Due to the low V concentration in the slag, its specific mineral form was not confirmed by XRD. However, the literature [39] suggests that in the presence of Mg and V under oxidizing conditions, various V compounds may form, including Mg_2_V_2_O_7_ and Mg_3_(VO_4_)_2_, which remain stable within slag systems at relevant temperatures. Calcium was identified as CaCO_3_ and in the form of various calcium silicates.

The diffraction pattern of the CH2 slag (Figure 7) indicates a broader spectrum of phases, particularly those containing Mg (gehlenite, merwinite, bredigite, spinel, and hercynite), as this slag contains a higher proportion of Mg compared to the CH1 slag. Dicalcium silicate is present similarly to the CH1 slag, and the small amount of Fe in the CH2 slag is likely bound in silicates or spinels. The presence of periclase was also detected in CH2. XRD and SEM-EDS analyses identified that Cr in the CH2 sample exists as an oxide or is bound primarily as a complex spinel (with Mg or Al) and as CrPO_4_.H_2_O. The XRD pattern indicated that Nb and V are bound in oxides with Fe and Ca.

The results of magnetic property measurements show that the EAF slag sample (CH1) exhibits magnetic characteristics with a coercive force of 4301 A/m, while the ferrochromium slag (CH2) behaves as a paramagnetic material. Density determination by helium pycnometry revealed that the CH1 slag reaches a value of 3.99 g/cm^3^, whereas a density of 3.27 g/cm^3^ was measured for the FeCr slag CH2.

### 2.4. Thermodynamic Study of the Proposal Thermochemical Activation Process

The primary objective of the proposed thermochemical activation process is the destruction of the stable spinel structures of the Cr- and V-bearing mineral phases in the original CH1 and CH2 slags to facilitate the release of Cr and V from the slag matrix. As indicated by mineralogical and phase analyses, Cr is primarily bound in compounds of the MgCr_2_O_4_ and FeCr_2_O_4_ types. Vanadium may potentially occur as Mg_2_V_2_O_7_ or Mg_3_(VO_4_)_2_ [39]; however, its low concentration in the analyzed sample precludes reliable detection of these specific phases.

The thermodynamic study of the slags thermochemical activation process was conducted using HSC Chemistry 10 [44]. This analysis predicted the reaction pathways of Cr- and V-containing spinel and oxide phases with alkaline reagents (NaOH and Na_2_O_2_, their combination, and KOH) within a temperature range of 200–800 °C. The standard Gibbs free energy changes (ΔG^0^) for the expected chemical reactions during the thermochemical activation process were calculated and are summarized in Table 2.

The results summarized in Table 2 indicate that chemical reactions leading to the transformation of stable Cr and V phases into more soluble forms are highly probable during the thermochemical activation process. In the case of Cr, this involves the conversion of stable Cr_2_O_3_, FeCr_2_O_4_, and MgCr_2_O_4_ phases into their respective chromates, specifically Na_2_CrO_4_ or K_2_CrO_4_ and K_2_Cr_2_O_7_. Analogously, V phases (represented primarily by Mg_2_V_2_O_7_ and Mg_3_(VO_4_)_2_) are transformed into soluble vanadates, predominantly in the forms of NaVO_3_, Na_3_VO_4_, Na_4_V_2_O_7_, or their potassium equivalents K_4_V_2_O_7_ and K_3_VO_4_. The formation of these salts is a crucial step in the thermochemical activation process; unlike the original spinel structures, these compounds are easily extractable into an aqueous solution during the subsequent leaching step.

The calculated ΔG^0^ values for these reactions are negative in all cases, even at a temperature of 200 °C, suggesting a high probability of a spontaneous process even at relatively low temperatures.

**Figure 8:**
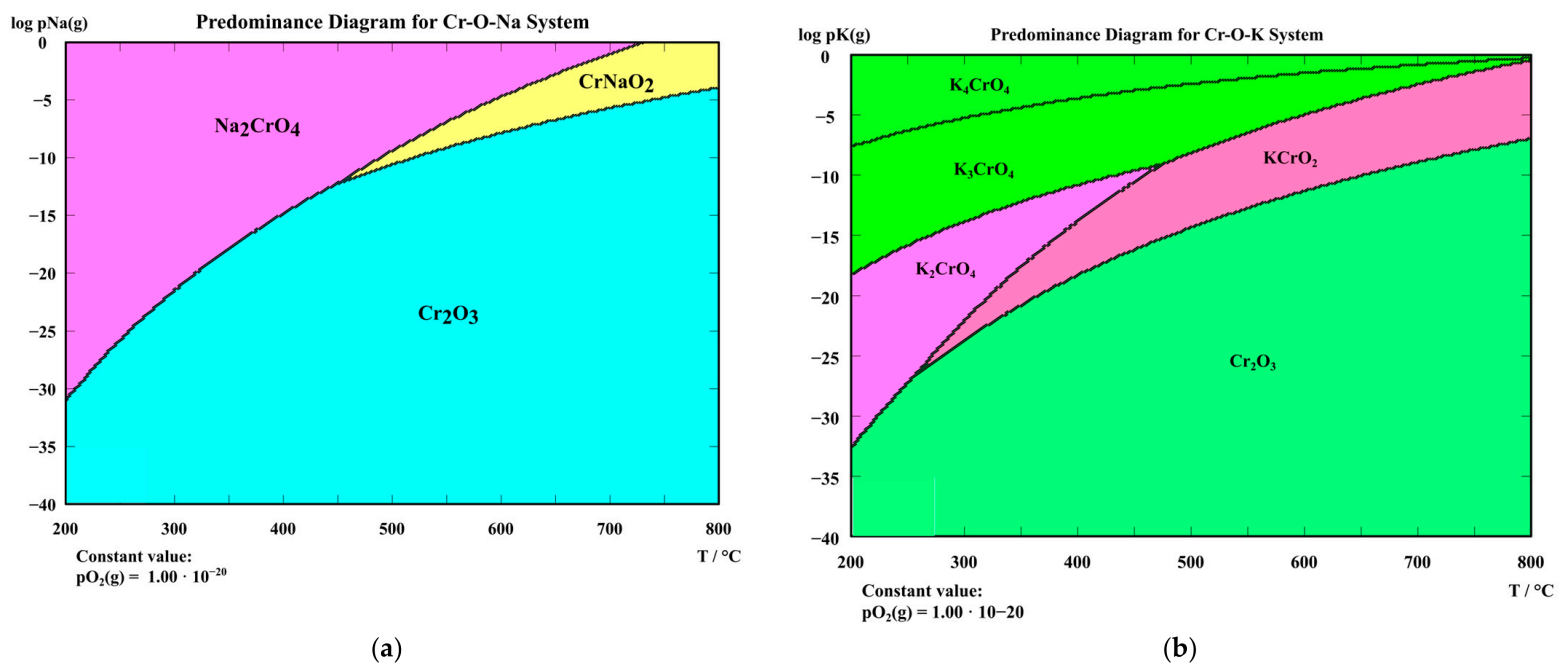
Predominance area diagram for (**a**) the Cr–O–Na system and (**b**) the Cr–O–K system as a function of temperature and Na/K chemical potential (calculated for pO_2(g)_ = 1.00 · 10^−20^ atm).

Furthermore, phase stability predominance diagrams (TPP–Temperature–Partial Pressure) were constructed using HSC Chemistry 10 [44] to predict the thermodynamic feasibility of the reactions and to identify the favorable conditions for the decomposition of the stable spinel structures. The phase boundaries and stability regions of Cr compounds in the Cr–O–Na and Cr–O–K systems, as a function of temperature and the chemical potential of sodium or potassium, are illustrated in Figure 8. They were calculated for a constant partial pressure of oxygen (pO_2(g)_ = 1.00 · 10^−20^ atm) over a temperature range of 200–800 °C.

As shown in Figure 8a, water-soluble Na_2_CrO_4_ is thermodynamically stable over a wide range of sodium activities, particularly at lower temperatures. With decreasing temperature, the stability boundary shifts toward lower log p_Na(g)_ values, indicating that the conversion of Cr into a leachable form is highly effective within the 200–600 °C range, even with smaller amounts of the sodium reagent. Although the log p_Na(g)_ axis formally represents gaseous sodium, in the thermodynamic model, it corresponds to the activity of the sodium reagent (e.g., NaOH or Na_2_O_2_). Thus, higher values represent a higher concentration of sodium ions in the reaction mixture. At temperatures above 500 °C, the stability region of the chromate gradually narrows, and the equilibrium shifts toward less soluble forms such as Cr_2_O_3_, or CrNaO_2_. This correlates with the observation that chromates form at low-to-moderate temperatures, whereas at higher temperatures, Cr^3+^ oxides (e.g., Cr_2_O_3_) persist, as they are thermodynamically more stable and less soluble in alkaline environments. This trend implies that high-temperature thermochemical activation (above 800 °C) leads to the reversion of Cr into less leachable forms, thereby hindering its subsequent extraction into solutions. This mechanism is thermodynamically supported by experimental studies, where phase composition at high temperatures shifts from chromate salts toward oxidic forms of Cr [45]. Furthermore, Figure 8a demonstrates that temperatures up to 600 °C provide a sufficiently wide thermodynamic “stability window” for the target product, allowing for high Cr yields at a lower process energy intensity. In the case of potassium reagents (e.g., KOH) (Figure 8b), the situation is analogous, with the diagram indicating the stability of K_2_CrO_4_ at lower temperatures. However, at higher temperatures and potassium activities, other forms of Cr compounds emerge (e.g., K_3_CrO_4_, K_4_CrO_4_), suggesting a more complex phase equilibrium compared to the sodium-based system.

**Figure 9:**
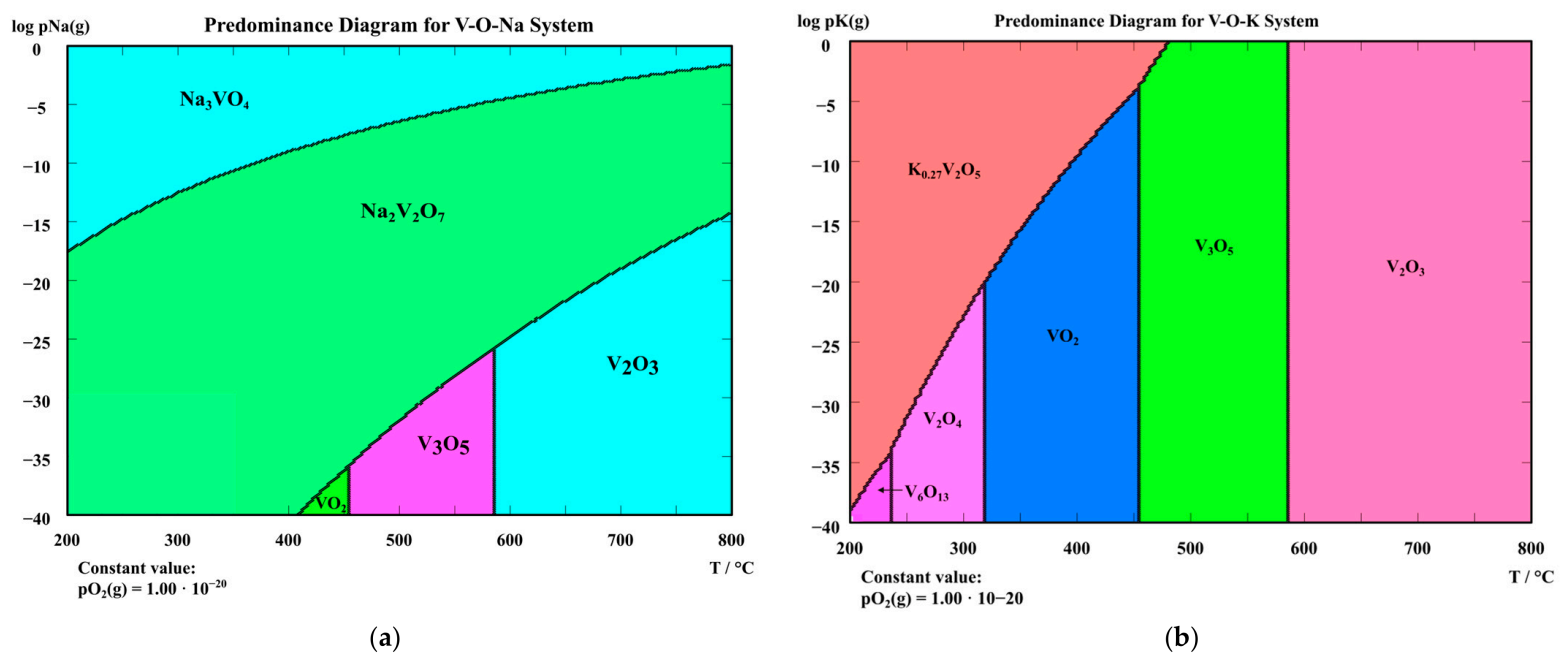
Predominance area diagram for (**a**) the V–O–Na system and (**b**) the V–O–K system as a function of temperature and Na/K chemical potential (calculated for pO_2_(g) = 1.00 · 10^−20^ atm).

The stability regions of V compounds in the V–O–Na and V–O–K systems as a function of temperature and Na/K chemical potential are illustrated via TPP predominance diagrams in Figure 9. In the V–O–Na system (Figure 9a), the predominance of water-soluble phases such as Na_3_VO_4_ and Na_2_V_2_O_7_ is evident across a wide range of sodium activities and temperatures up to approximately 500–600 °C. At temperatures exceeding 600 °C, the stability field shifts toward insoluble vanadium oxides (V_3_O_5_, V_2_O_3_), confirming that lower temperatures are favorable for V recovery. In the V–O–K system (Figure 9b), a similar trend is observed, where the soluble potassium–vanadium phase (K_0.27_V_2_O_5_) is stable at lower temperatures and higher potassium activities. However, as the temperature increases, the system transitions rapidly toward various lower-valence vanadium oxides (VO_2_, V_3_O_5_, V_2_O_3_), indicating that the stability field of the soluble phase contracts more significantly with temperature compared to the Na system. These diagrams confirm that effective thermochemical activation for V recovery should be conducted at temperatures below 600 °C to prevent the formation of refractory oxidic phases.

### 2.5. Thermochemical Activation Experiments Followed by Leaching

The input materials for the experiments were samples CH1 and CH2 with the composition specified in Table 1**,** ground to a particle size of 0.090–0.125 mm. Prior to thermochemical activation, the reaction mixture consisting of the slag and the respective reagent was thoroughly homogenized and placed in a porcelain crucible. The reagent dosage and slag-to-reagent ratio were determined based on stoichiometric calculations, with excess reagent utilized to ensure complete reaction of NaOH with the primary slag matrix components (Fe, Si, Ca, Mg, and Al oxides). Subsequently, a precisely defined amount of deionized water (16 mL) was added to the mixture to achieve a suitable porous structure of the product and to facilitate its subsequent quantitative releasing from the crucible. The crucible was placed in a muffle furnace (Ht40AL, LH 30/13, LAC, Ltd. Brno, Czech Republic) preheated to the target temperature. Upon completion of the thermochemical activation process, the resulting activated slag was crushed in an agate mortar. A representative sample of the product was collected for chemical analysis and XRD phase analysis.

Leaching of the CH1 and CH2 activated slag was carried out in a standard glass apparatus using deionized water at 60 °C, with stirring at 500 rpm. The leaching experiments were performed at liquid-to-solid (L:S) ratios of 20 and 40, depending on the specific experimental setup. The initial sample mass was 10 g, with leaching medium volumes of 200 mL and 400 mL, respectively. While the standard duration for leaching was set to 60 min, in selected kinetic experiments, solution samples (10 mL) were withdrawn at predetermined intervals to monitor the extraction progress. In specific cases for the CH2 slag, leaching was also conducted in 3 M H_2_SO_4_ to compare the extraction efficiency. After 60 min, the final leachate was filtered, and the concentrations of Cr, V, and accompanying matrix elements (Fe, Ca, Si, Mg) were determined by HR-CS FAAS. The obtained leaching residue was dried to a constant weight at 105 °C for 4 h and subsequently subjected to total sample digestion followed by HR-CS FAAS, while selected samples were also characterized by XRD analysis. All experiments were performed in triplicate. The results are presented as the mean value ± standard deviation (SD). Error bars in the figures represent the SD of these independent measurements (*n = 3*).

The metal extraction efficiency *(E)* was calculated according to Equation (31), defined as the ratio of the mass of the metal recovered in the leachate to the total initial mass of the metal present in the slag:

(31) $$E=\frac{C_{0}\cdot V}{m_{slag}\cdot \;w}\;.\;100\;[\%]$$

where *C*_o_ is the metal concentration in the leachate (mg/L); *V* is the volume of the leachate (*L*); *m_slag_* is the mass of the initial slag sample (g); and *w* is the weight fraction of the metal in the original slag (mg/g).

#### 2.5.1. Effect of Various Reagents During Thermal Activation on Cr and V Leaching

The following reagents and their mixtures were initially selected for the slag thermochemical activation experiments: NaOH, KOH, NaOH–Na_2_O_2_, NaOH–NaNO_3_, Na_2_O_2_, NaOH–NaCl, KOH–K_2_CO_3_, H_3_BO_3_, NaH_2_PO_4_·xH_2_O, (NH_4_)_2_SO_4_–KHSO_4_, K_2_S_2_O_5_, and K_2_S_2_O_7_. Preliminary experiments conducted on the CH1 slag at temperatures of 300–500 °C for 1–2 h showed that the use of K_2_S_2_O_5_, K_2_S_2_O_7_, H_3_BO_3_, Na_2_SO_4_·10H_2_O, and NaH_2_PO_4_·xH_2_O resulted in practically zero or very low leaching yields of both Cr and V during subsequent water leaching. In these cases, the leaching was primarily limited to Ca and partially Si, indicating that these reagents were unable to effectively decompose the inert Cr and V spinel phases present in the CH1 slag. Furthermore, in the sulphate systems (K_2_S_2_O_7_, Na_2_SO_4_·10H_2_O), a massive transfer of Mg into the solution was recorded (up to 975 µg/mL Mg). For these reasons, these reagents were excluded from further experiments with the CH1 slag, and attention was focused primarily on alkaline thermochemical activation systems exhibiting higher affinity and reactivity toward the target metals.

Thermochemical activation experiments for both types of slag focused on monitoring the effects of temperature and thermochemical activation time. The experiments were performed in a temperature range from 250 °C to 800 °C, with thermochemical activation times from 30 min to 3 h. The thermochemical activation temperatures were selected with respect to the melting points of the individual reaction systems reported in the literature [36,42,43,46,47]. An overview of the thermochemical activation experiments that led to the thermochemical activation of both slags, followed by water leaching of Cr and V, is provided in Table 3. Water leaching was carried out under constant conditions: 10 g of activated slag (CH1 or CH2), 200 mL H_2_O (L:S ratio = 20), T = 60 °C, stirring at 500 rpm, and t = 1 h.

In contrast to the CH1 slag, where the use of most sulphate thermochemical activation reagents did not lead to remarkable extraction of Cr and V during subsequent water leaching, the CH2 slag roasted with K_2_S_2_O_5_, K_2_S_2_O_7_ and Na_2_SO_4_·10H_2_O achieved higher efficiency when combined with subsequent acidic leaching in H_2_SO_4_. The conditions for the thermochemical activation experiments of the CH2 slag using sulphate reagents, followed by acidic leaching of the activated slag (leaching conditions: 10 g of activated CH2 slag, 400 mL 3 M H_2_SO_4_, L:S = 40, T = 60 °C, 300 rpm, t = 1 h), are summarized in Table 4.

#### 2.5.2. Study of Thermochemical Activation Parameters for the NaOH System

Based on the results of the experiments with various reagents, NaOH was selected as the reference alkaline thermochemical activation agent for subsequent experiments. These were focused on monitoring the effects of the temperature and reagent dosage on the leaching yields of Cr and V from both CH1 and CH2 slags.

These experiments were conducted within a temperature range of 200–600 °C, which was selected based on thermodynamic calculations (Table 2) and available literature data [38,48,49] The effect of NaOH dosage was investigated in the range of 4, 8, 12, and 16 g, while other experimental conditions—slag mass and activation time—remained constant (12 g of CH1/CH2 slag, t = 2 h). Upon completion of the thermochemical activation process, the resulting activated products (10 g samples) were subjected to leaching in deionized water under standardized constant conditions (L:S = 20, T = 60 °C, 500 rpm, t = 1 h). The recovery yields of the target metals were calculated based on their initial content in the activated slags. The objective of these experiments was to assess the impact of individual process parameters and to establish suitable conditions for the alkaline treatment of CH1 and CH2 slags using NaOH.

## 3. Results and Discussion

### 3.1. Leaching Results for Slags Thermochemically Activated with Various Reagents

**Figure 10:**
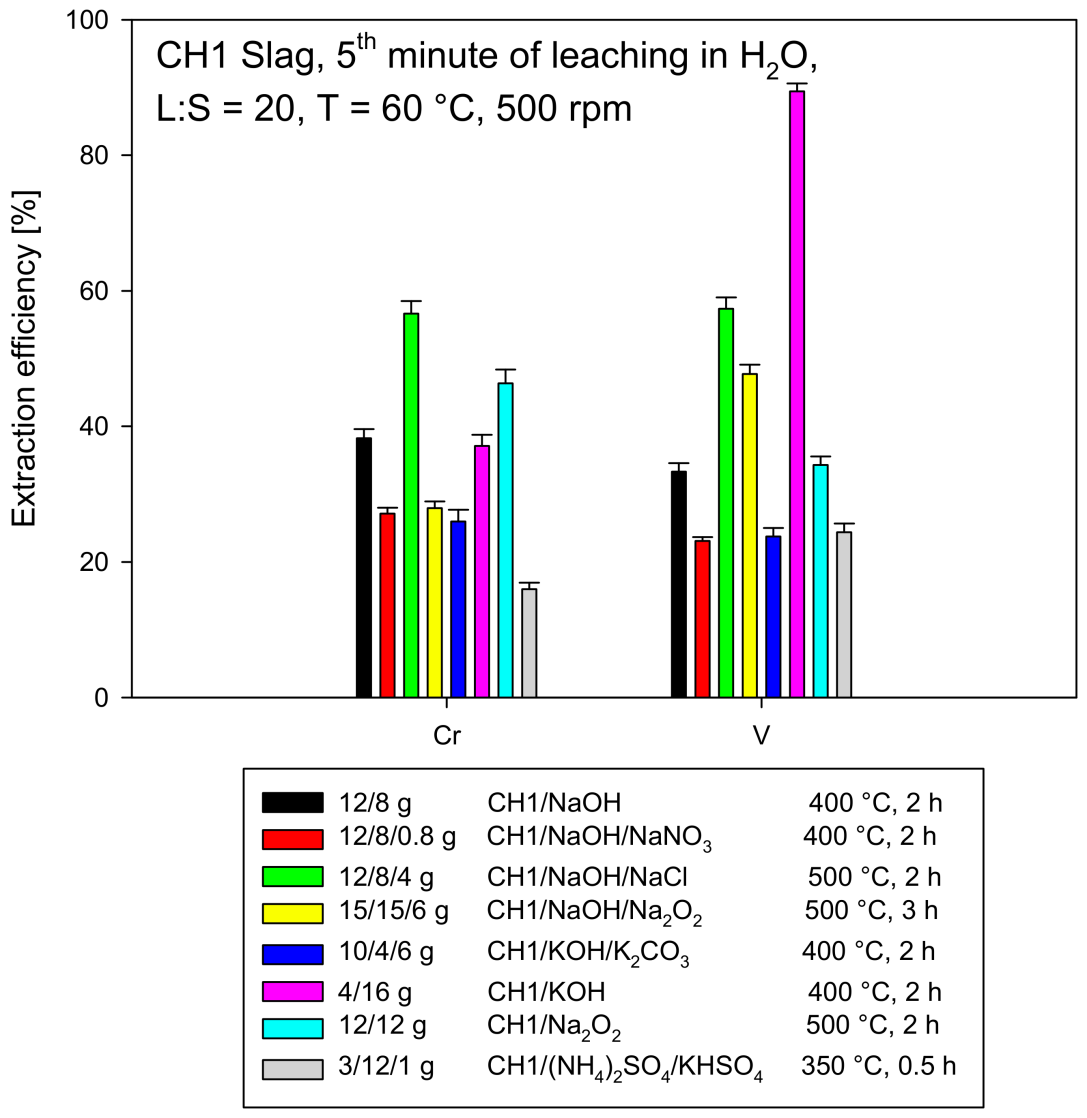
Metal extraction from CH1 slag: impact of thermochemical activation conditions on subsequent water leaching efficiency.

**Figure 11:**
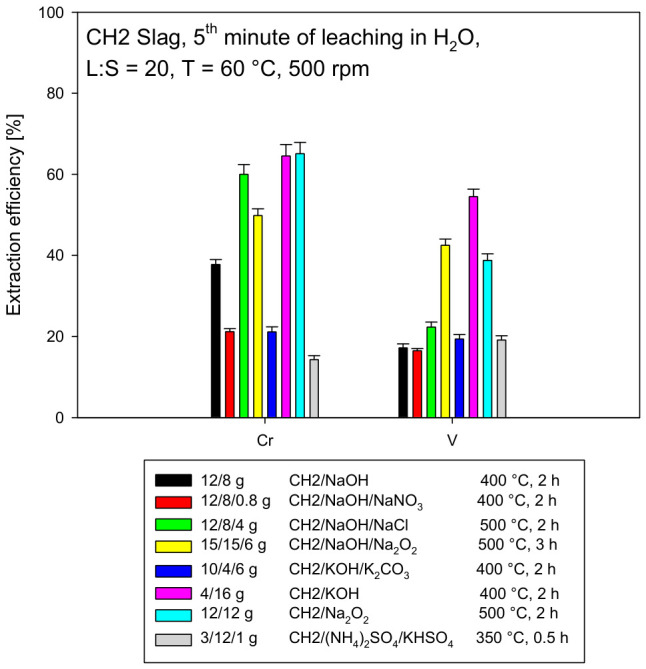
Metal extraction from CH2 slag: impact of thermochemical activation conditions on subsequent water leaching efficiency.

The results of the thermochemical activation (experimental sets 1–8) are presented in Figure 10 for the CH1 slag and Figure 11 for the CH2 slag. Although the subsequent water leaching was conducted for a total duration of 1 h, the concentrations of Cr and V in the leachate reached their maximum within 5 min and remained practically unchanged thereafter. Consequently, the results presented hereafter focus on the leaching yields obtained at the 5th minute, representing the maximum extraction efficiency achieved under the specified conditions.

While these results demonstrate rapid and efficient metal recovery, we acknowledge that investigating long-term stability is a crucial step for future industrialization. The complex nature of the slag matrix, the specific composition of thermochemically activated products, and the presence of iron precipitates in leaching residues, sorbent-like structures, and fine-grained matter may influence potential re-adsorption or re-precipitation phenomena.

In terms of Cr extraction efficiency, the NaOH–NaCl combination (experimental set No. 3) at 500 °C proved to be the most effective system for CH1 with a yield of 56.6% Cr. For CH2, the maximum yield of 65% Cr was achieved using a mixture of NaOH-Na_2_O_2_ (experimental set No. 7). A similar Cr extraction efficiency for CH2 was observed using KOH (experimental set No. 6), while the NaOH–NaCl system (experimental set No. 3) yielded quite comparable results (≈60%).

Regarding the V extraction efficiency, the optimal conditions for both CH1 and CH2 slags were achieved using KOH at 400 °C (experimental set No. 7), resulting in extraction efficiencies of 89% and approximately 55%, respectively.

In contrast, the addition of the oxidizing agent NaNO_3_ to NaOH (experimental set No. 2) did not increase the efficiency of thermochemical activation, as evidenced by the extraction efficiency of Cr and V for either of the slags. Instead, it led to a decrease in Cr yield for both slags in comparison to the single NaOH system (experimental set No. 1), suggesting a suppressive influence of NaNO_3_ on the effective phase conversion of Cr into a leachable form in the presence of NaOH.

The lowest leaching yields for both Cr and V were recorded using the (NH_4_)_2_SO_4_/KHSO_4_ mixture (experimental set No. 8). For the CH1 slag, the extraction efficiencies of Cr and V reached 16.0% and 24.4%, respectively, while for the CH2 slag, the values were even lower, at 14.3% for Cr and 19.1% for V.

**Figure 12:**
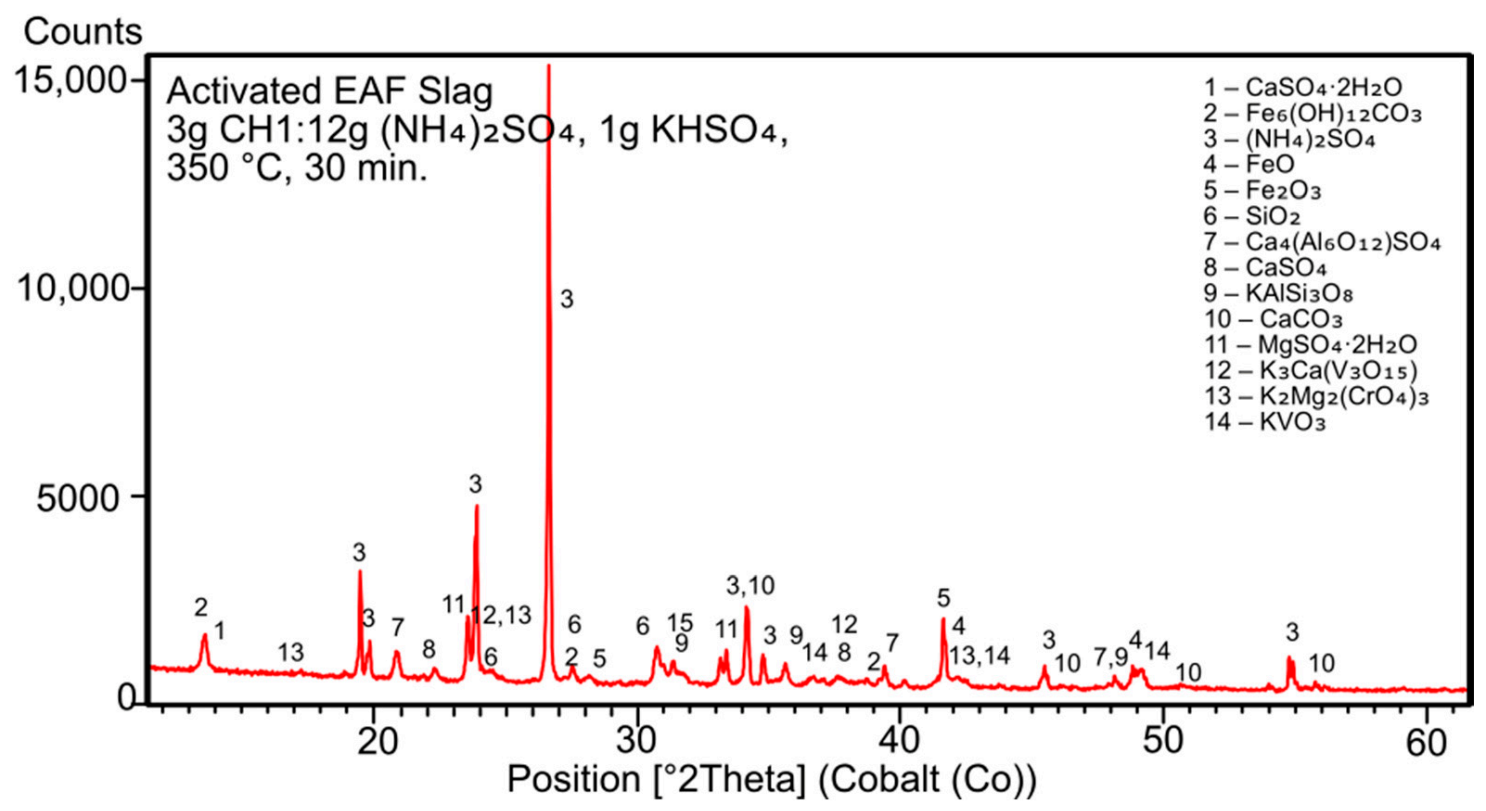
XRD pattern of activated CH1 sample in the acid thermochemical activation system with (NH_4_)_2_SO_4_/KHSO_4_.

The poor efficiency of this system is primarily a direct consequence of the insufficient thermochemical activation temperature, which failed to provide enough energy to initiate the breakdown of the stable spinel structures by the sulphates. As evidenced by the phase analysis of CH1 slag (XRD pattern in Figure 12), a significant portion of the (NH_4_)_2_SO_4_ reagent remained thermally inactive or did not participate in the intended reactions, with its characteristic diffraction lines still clearly present. This indicates that the reagent did not contribute to the formation of soluble Cr and V species under these conditions. While KHSO_4_ showed some degree of participation, the system’s reactivity was largely diverted by the high content of matrix elements such as Ca, Mg, and Si. One of the contributing factors was the preferential reaction of sulphates with Ca to form anhydrite (CaSO_4_) according to reaction (32):

*CaCO*_3_* + (NH*_4_*)*_2_*SO*_4_* = CaSO*_4_* + 2NH*_3_*(g) + CO*_2_*(g) + H*_2_*O(g), ΔG*^0^_773.15_* = −145* [kJ] (32)

In addition to Cr and V, the presence of accompanying elements—specifically Si, Ca, Fe, and Mg—was analyzed in the final leachates to assess the selectivity of individual thermal activation and leaching systems. The weight loss of the samples during leaching ranged from 30–50% (CH1) and 20–50% (CH2), and in the case of KOH, it reached 60% by weight for CH1 and 70% for CH2. These losses are related to and, to a certain extent, correlate with the mass of the reagent used and the leachable phases formed during activation, which confirms the process selectivity and matrix stability under the given conditions.

Despite 1. leachates. The Fe, which was present in the input CH1 sample in the form of FeO and Fe_3_O_4_, was converted to NaFeO_2_ during thermochemical activation according to reactions (33) and (34):

*4FeO + 4NaOH + O*_2*(g)*_* = 4NaFeO*_2_* + 2H*_2_*O_(g)_, ΔG*^0^_773.15_* = −472* kJ (33)

*4Fe*_3_*O*_4_* + 12NaOH + O*_2*(g)*_* = 12NaFeO*_2_* + 6H*_2_*O_(g)_, ΔG*^0^_773.15_* = −551* kJ (34)

The absence of Fe in the leachates after water leaching of the CH1 slag is attributed to the spontaneous hydrolysis of unstable NaFeO_2_ (as shown in reactions (35) and (36)), which results in the partial precipitation of Fe during leaching and continues during the cooling of the leachate.

*NaFeO*_2_* + 2H*_2_*O = Fe(OH)*_3_* + NaOH, ΔG*^0^_333.15_* = 31.733* kJ (35)

*NaFeO*_2_* + H*_2_*O = FeOOH + NaOH, ΔG*^0^_333.15_* = 9.997* kJ (36)

Although theoretical assumptions and software calculations based on the positive *ΔG^0^* value for reactions (35) and (36) suggest that the reaction of NaFeO_2_ with water should not occur, experimental results confirm its spontaneous hydrolysis. This phenomenon is likely caused by the zero initial activity of NaOH in water and the high stability of Fe hydroxides and oxyhydroxides in water, which possess extremely low solubility products (e.g., K_sp_ Fe(OH)_3_ = 2.79·10^−39^) [50].

The concentration of silicon in the leachates of NaOH-activated samples after water leaching was relatively low, with a maximum of 7% Si leached from CH1 and ≈1% from CH2. Higher Si yields (32%) were achieved when using a significant excess of KOH (16 g) to activate the CH1 slag. With a lower KOH ratio or when using a KOH–K_2_CO_3_ combination, the Si yields were comparable to the NaOH systems, reaching ≈ 6% for CH1 and up to 2% for CH2.

Based on the stated results, it can be concluded that alkaline thermochemical activation and leaching systems enable high Cr and V yields while simultaneously ensuring the low leachability of both slag matrices.

**Figure 13:**
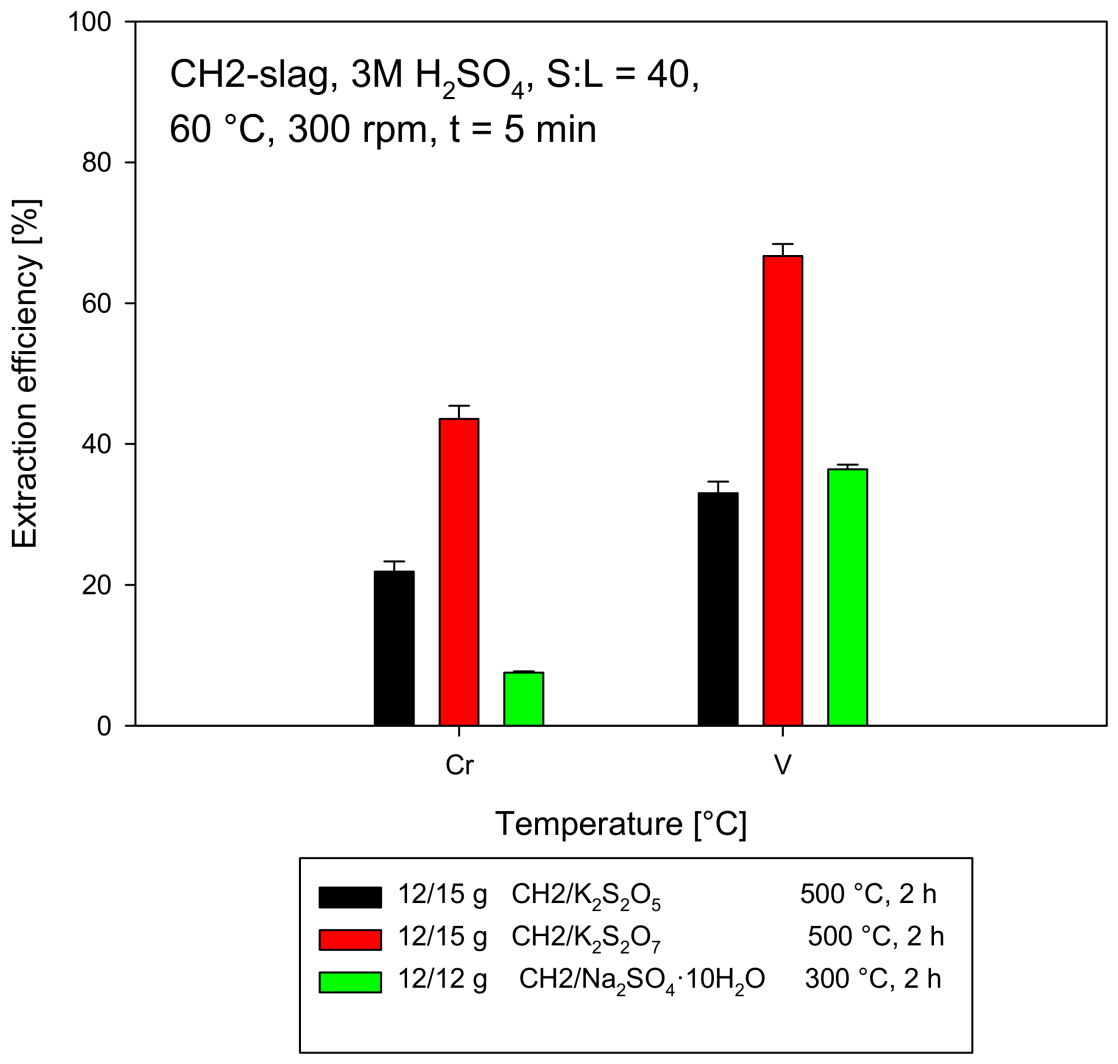
Metal extraction from CH2 slag: influence of thermochemical activation conditions on subsequent leaching efficiency in 3 M H_2_SO_4_.

In Figure 13, the results of the thermochemical activation of the CH2 slag with a subsequent sulphuric acid leaching are presented. The samples were subjected to thermochemical activation using sulphate reagents (K_2_S_2_O_5_, K_2_S_2_O_7_, and Na_2_SO_4_·10H_2_O), followed by leaching in 3 M H_2_SO_4_.

Leaching of the CH2 slag, previously activated with the acidic reagent K_2_S_2_O_5_, resulted in extraction efficiencies of 21.9% for Cr and 33.0% for V after 5 min of leaching in 3 M H_2_SO_4_. Even higher values were recorded during the use of the K_2_S_2_O_7_ reagent, where the Cr yield reached 43.56% and the V yield reached 66.7% during the same leaching period. The lowest yields among the monitored sulphate thermochemical activation reagents were observed for Na_2_SO_4_.10H_2_O, representing 7.5% for Cr and 36.4% for V after 5 min of leaching. While thermochemical activation of the CH2 slag with sulphate reagents provides interesting results, the overall Cr extraction efficiency remains below the levels achieved by alkaline systems. A further disadvantage of this thermochemical activation process is that, in addition to the target Cr and V, it results in the remarkable leaching of matrix elements such as Mg and Ca.

### 3.2. Results of Thermochemical Activation Parameters Study for the NaOH System

In accordance with the experimental design described in the Materials and Methods, NaOH was used as the reference alkaline agent for further study. This investigation examined the influence of thermochemical activation temperature and the amount of reagent on the extraction yields of Cr and V from CH1 and CH2 slags. Consequently, the following section analyzes two key factors:

- Thermochemical activation temperature, which influences the thermodynamic stability of the spinel phases within the slag;
- NaOH dosage, with the aim of determining the optimal amount of reagent for maximum Cr and V extraction while simultaneously limiting the leaching of Si and Ca.

#### 3.2.1. Effect of Thermochemical Activation Temperature for the NaOH System

**Figure 14:**
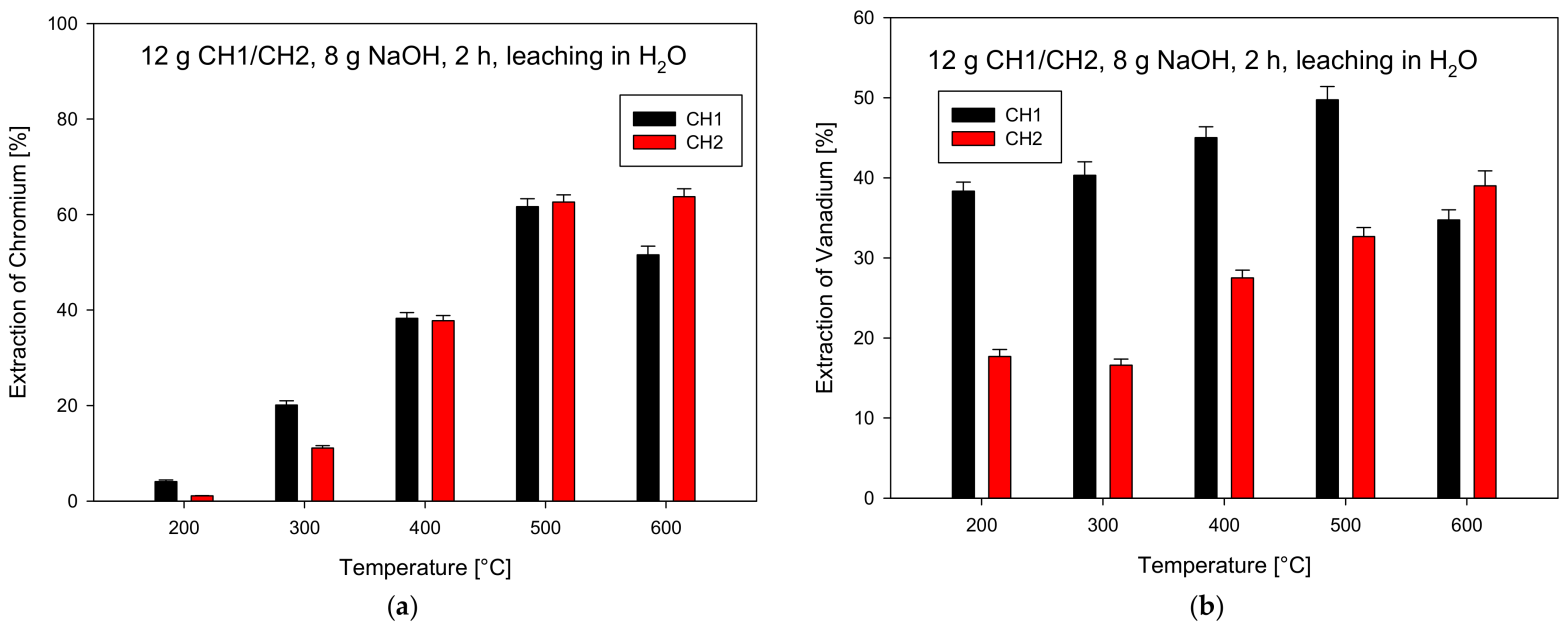
Effect of thermochemical activation temperature on the extraction efficiency of (**a**) Cr and (**b**) V from CH1 and CH2 using 8 g NaOH (leaching time: 5 min).

The influence of thermochemical activation temperature on Cr and V extraction efficiencies during the thermochemical activation of 12 g of CH1/CH2 slag (8 g NaOH, 2 h activation time, followed by 5 min of water leaching) is illustrated in Figure 14.

Figure 14 indicates that temperature is a key factor in thermochemical activation and significantly influences the extraction of both Cr and V. For both types of slag, the dependence of yield on temperature exhibits a rising trend in the process of overcoming the energy barrier required to disrupt either the Fe spinel (CH1) or the stable silicate structures (CH2), thereby enabling the reactions to form sodium compounds of Fe, Cr and V.

**Figure 15:**
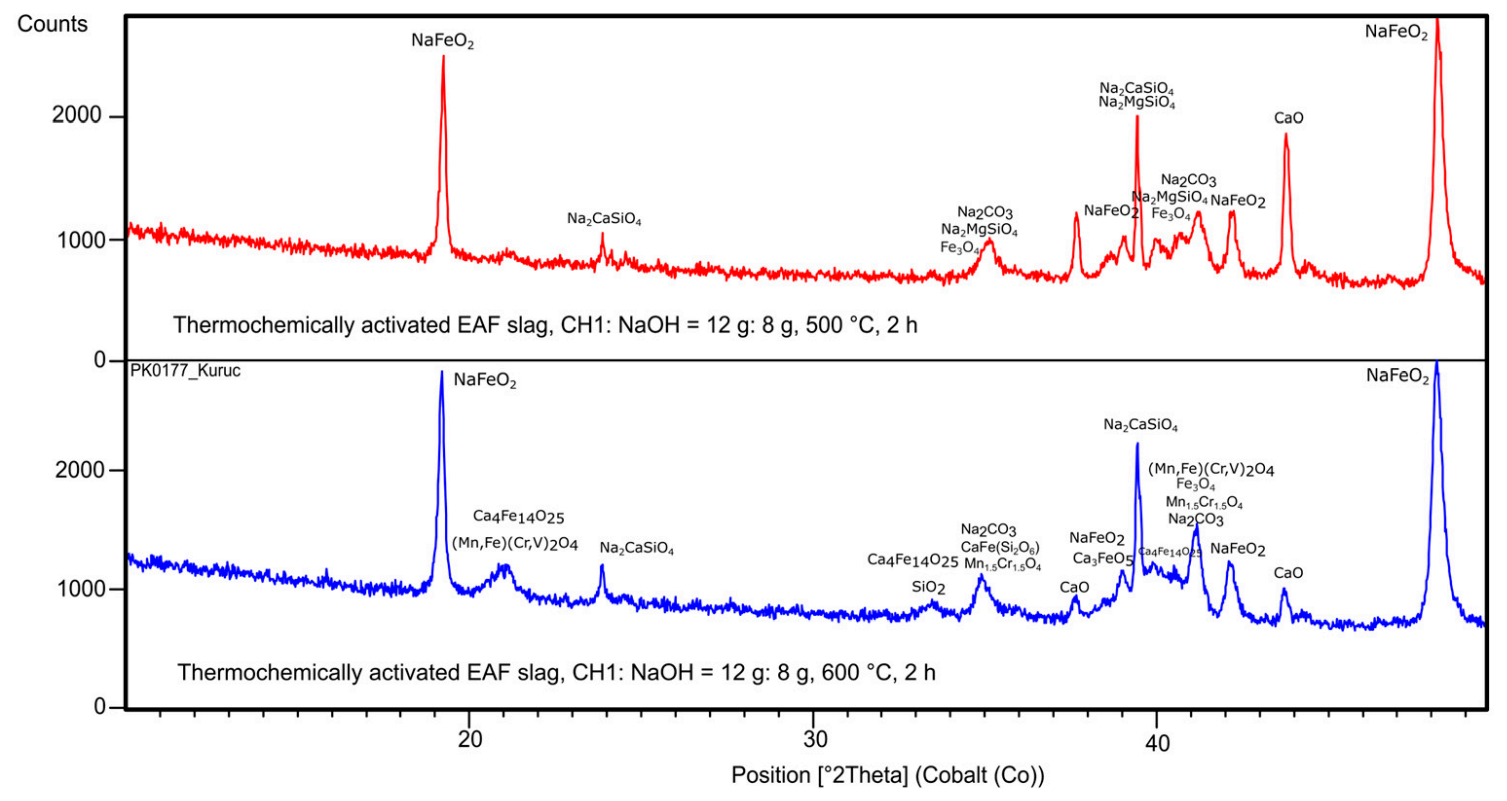
Comparison of XRD diffractograms of the activated CH1 slag at 500 °C and 600 °C under identical experimental conditions.

The increase in Cr yield (Figure 14a) within the temperature range of 300–500 °C is likely a direct consequence of the formation of eutectic mixtures and the transition of the system into a low-melt-viscosity region. Although the melting point of pure NaOH is approximately 318 °C, eutectic formation occurs between the reactant and the matrix components in a real slag system. These mixtures lower the melting point of the system, enabling the formed melt to improve the penetration of the reagent into inert phases [51]. The Cr and V yields for CH1 showed a notable drop at a temperature of 600 °C. This phenomenon can be explained by the different phases formed in the slag when it is activated at 600 °C compared to 500 °C. As can be seen in the XRD pattern of CH1 slag activated at 600 °C (Figure 15, blue pattern), more phases based on Ca, Fe and Si (e.g., Ca_4_Fe_14_O_25_, Ca_3_FeO_5_ and CaFe(Si_2_O_6_)) were identified. Additionally, the Mn-Cr spinel (Mn_1_._5_Cr_1_._5_O_4_) phase, as well as the complex chromite spinel binding of Cr and V (such as (Mn,Fe)(Cr,V)_2_O_4_), was also identified at 600 °C. These phases are thermodynamically more stable at this temperature. Under these conditions, the FeO and Fe_3_O_4_ phases are promoted to react preferentially with Cr and V, intercalating them into their structures. The spinel phase Mn_1_._5_Cr_1_._5_O_4_ represents the thermally affected, Mn-enriched structure of the spinel MnCr_2_O_4_ in the presence of oxygen [52]. None of these phases reacted with NaOH, resulting in a drop in the leaching extraction of Cr and V in water, as can be seen in Figure 14. It is likely that these phases require different thermal activation and leaching conditions, which need to be studied further.

In contrast to the CH_1_ slag, the CH_2_ slag did not exhibit a decrease in metal extraction as the thermochemical activation temperature increased to 600 °C; instead, the extraction efficiency gradually increased. XRD analysis of CH_2_ slag activated at 600 °C demonstrated a phase composition similar to that observed at 500 °C, confirming the formation of the NaCrO_4_ phase. Although the XRD pattern of the activated CH_1_ (Figure 15) slag did not reveal soluble Cr/V phases due to their low concentration, it is expected that similar phases were formed during its activation [53]. The lower content of Fe phases in the CH_2_ slag resulted in less complex XRD patterns characterized by minimal peak overlap. The absence of overlapping diffraction peaks in the XRD patterns facilitated the detection of minor Cr- and V-based phases in the activated CH_2_ slag. Consequently, it became possible to distinguish these minor phases.

In the case of V (Figure 14b), the influence of temperature on extraction is less critical than for Cr in CH1 and CH2, with only minor differences in extraction visible as the temperature rises to 400 °C. The Cr phases are gradually decomposed, while the V species seem to be thermochemically activated to some extent at low temperatures. As shown in Figure 14b), V extraction efficiency is around 40% for CH1 and 20% for CH2 at 200 °C, whereas for Cr, it is approximately 5% for CH1 and 2% for CH2. Vanadium extraction efficiency slightly increases at temperatures of 400 °C and 500 °C in CH1, probably due to the presence of molten NaOH, which increases the system’s reactivity, reaction kinetics, and transport phenomena in the liquid phase. Liquid NaOH enables the active disruption of the magnetite lattice and other phases in which V is likely to be present. This behavior could also indicate the presence of V species that react differently during activation, or phases of V that are leachable to different extents. A decrease in V extraction is also observed in CH1 slag activated at 600 °C, similarly to Cr (Figure 14a). Furthermore, the leaching efficiency of both metals is influenced by the formation of high-viscosity silicate species—identified by XRD analysis (Figure 15)—thereby hindering the diffusion process and limiting the overall leaching kinetics [54]. However, the overall extraction efficiencies of V and Cr for both slags show that complete leaching would require the total decomposition of spinel (in CH1) or the silicate structure (in CH2).

#### 3.2.2. Effect of NaOH Dosage

**Figure 16:**
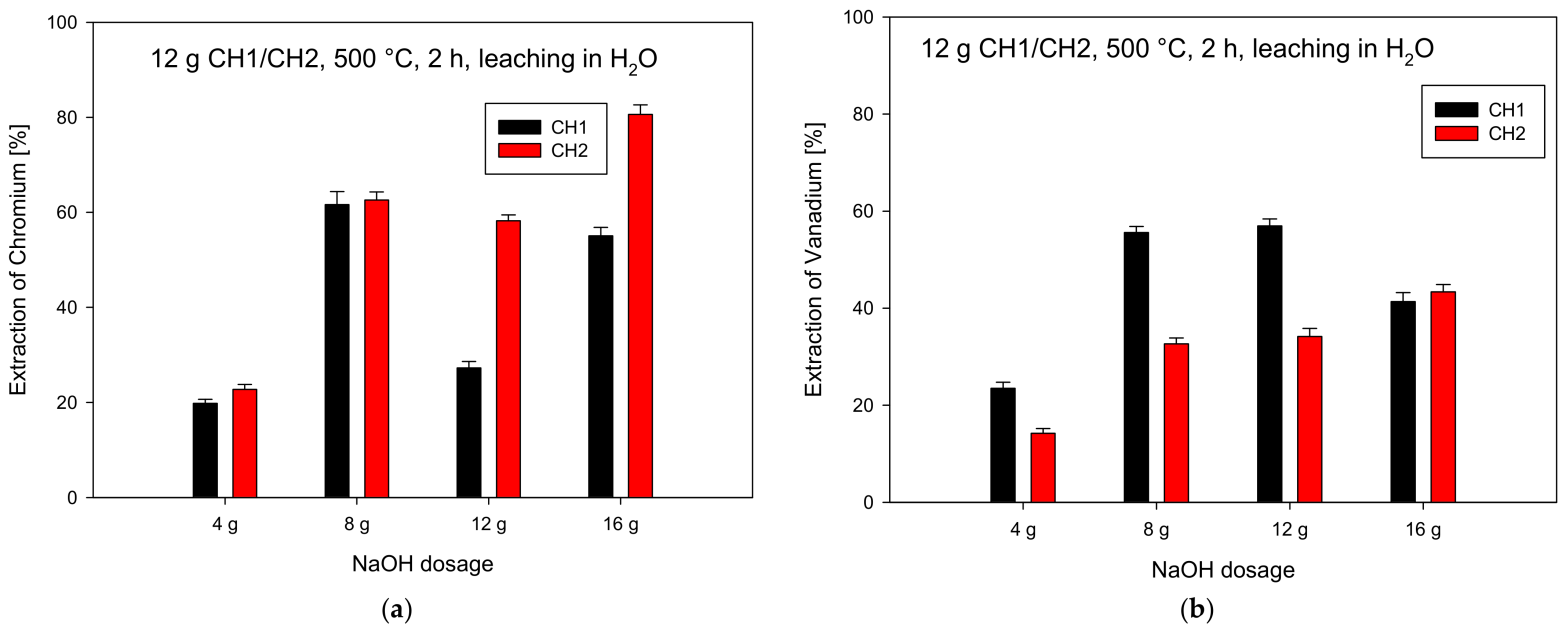
Effect of NaOH dosage during thermochemical activation on the subsequent extraction efficiency of (**a**) Cr and (**b**) V from CH1 and CH2 (500 °C, 2 h; leaching in H_2_O, 5 min).

The influence of NaOH dosage on Cr and V extraction efficiencies during the thermochemical activation of 12 g of CH1/CH2 slag (500 °C, 2 h activation time, followed by 5 min water leaching) is illustrated in Figure 16.

Figure 16a indicates that the dependence of Cr yield on the amount of alkaline reagent is non-linear and varies according to the slag type and NaOH dosage. In the case of the CH1 slag, the Cr yields initially increased substantially with rising amounts of NaOH, reaching a maximum (61.6%) at a NaOH dosage of 8 g. A further increase in the alkaline reagent to 12 g led to a decrease in yield; however, at 16 g of NaOH, an increase in Cr extraction was recorded, though it remained lower than at the optimal 8 g dosage. The decrease in Cr yield from the CH1 slag at excess NaOH levels (above 8 g) can be explained, in agreement with other authors [36], by the preferential reaction of the alkaline reagent with the silicate matrix of the slag.

**Figure 17:**
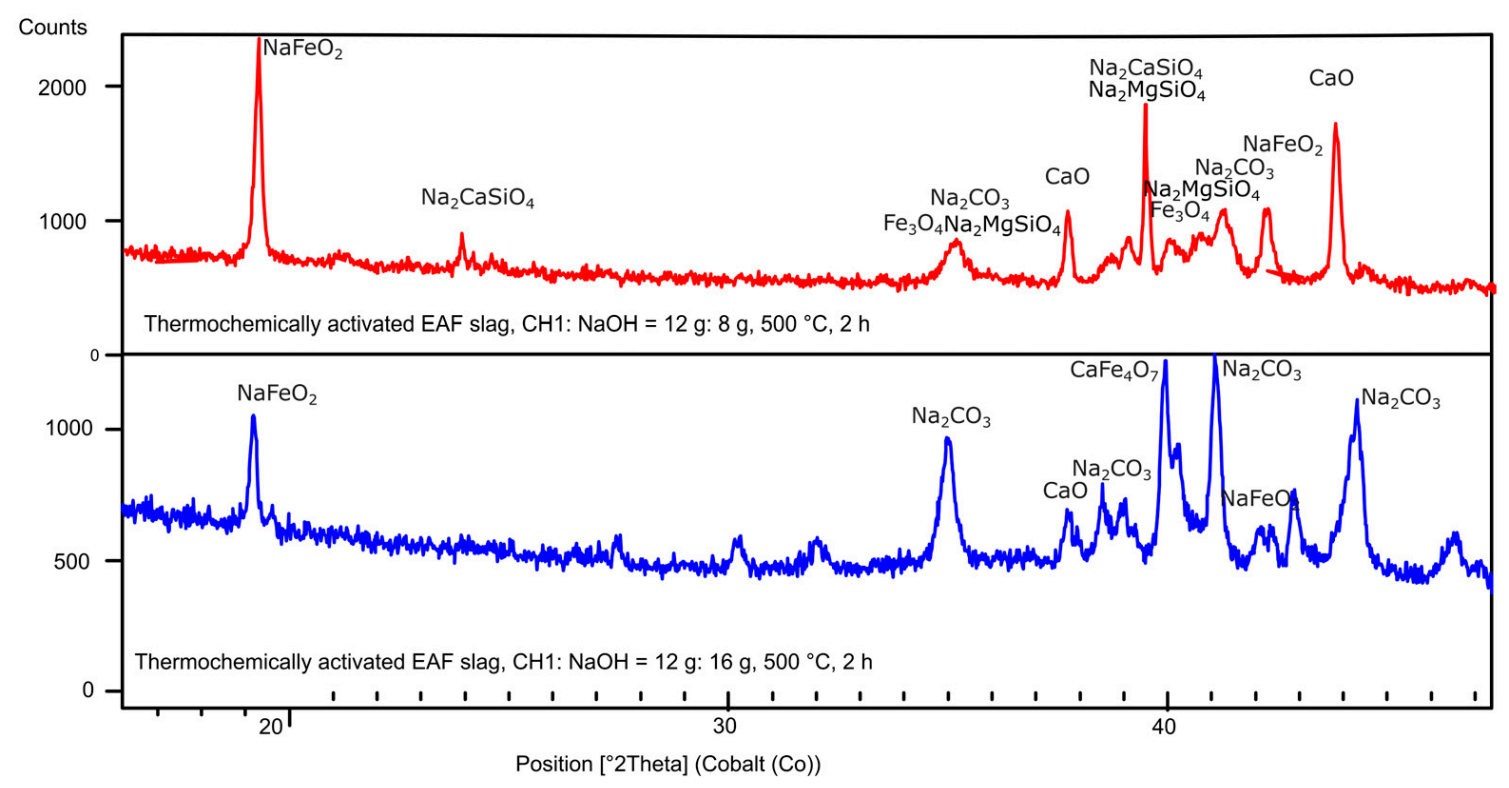
Comparison of XRD diffractograms of CH1 thermochemically activated samples prepared with different NaOH dosages (8 g and 16 g).

**Figure 18:**
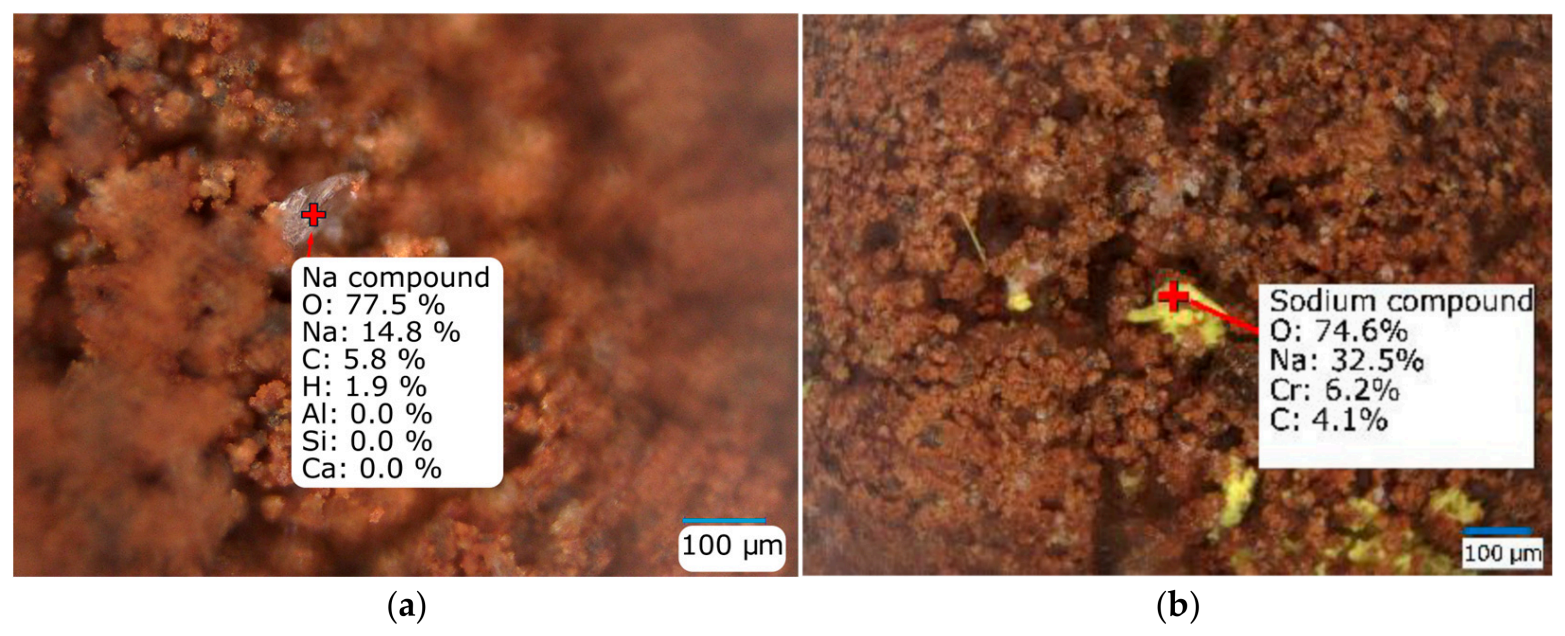
Optical micrograph of activated CH1 slag (8 g NaOH, 500 °C, 2 h) with LIBS point anal-ysis indicating: (**a**) the presence of Na_2_CO_3_ particle alongside other phases; (**b**) yellow particles Na_2_CrO_4_ together with Na_2_CO_3_.

By comparing the XRD patterns of samples prepared with 8 g and 16 g of NaOH (Figure 17), it is evident that the phase composition of activated CH1 slag at 500 °C is significantly influenced by the NaOH dosage. While 8 g of NaOH facilitates the formation of calcium silicates and NaFeO_2_, a higher NaOH content (16 g) promotes the preferential synthesis of calcium ferrite (CaFe_4_O_7_) and the creation of Na_2_CO_3_ and reduces the formation of NaFeO_2_. Furthermore, the presence of Na_2_CO_3_ in the samples prepared with 8 g of NaOH was confirmed by LIBS point analysis (Figure 18a), providing direct evidence for the “in situ” transformation of the activation reagent.

This shift suggests that the system’s phase equilibrium during the thermochemical process is governed by the dosage of NaOH and competing interactions: (a) CaO-CaCO_3_-Ca(OH)_2_, (b) NaOH-Na_2_CO_3_, and (c) FeO-CaO. Exchange reactions between CaCO_3_ and NaOH (37) and (38) are crucial, as they regulate the availability of “free” NaOH necessary for spinel disruption (Fe_3_O_4_, FeCr_2_O_4_). The presence of CaO as a reaction product of (37) was confirmed by XRD analysis and is well-supported by the literature [55].

*CaCO*_3_* + 2NaOH = Na*_2_*CO*_3_* + CaO + H*_2_*O_(g)_, ΔG*^0^_773.15_* = −37.082* kJ (37)

*CaCO*_3_* + 2NaOH = Na*_2_*CO*_3_* + Ca(OH)*_2_*, ΔG*^0^_773.15_* = −39.307* kJ (38)

The mineralogical composition is highly sensitive to the NaOH-to-slag ratio: at lower ratios, CaCO_3_ reacts preferentially with NaOH to form CaO and calcium–sodium silicates (e.g., Na_2_CaSiO_4_). Conversely, at higher NaOH dosages, the formation of CaO is suppressed in favor of Na_2_CO_3_ and calcium ferrite phases (39).

*4FeO + 2CaO + O*_2*(g)*_* = 2CaO·Fe*_2_*O*_3_*, ΔG*^0^_773.15_* = −447.363* kJ (39)

Thus, the CaCO_3_ content in the slag acts as a critical parameter, where high levels can inadvertently hinder Cr and V extraction by consuming the active NaOH reagent.

There are a few possible ways to remove calcium carbonate from the slag, thereby enhancing its thermochemical activation and improving the efficiency with which the activation reagent is utilized. These options include, for example, thermal pretreatment methods such as calcination or pyrolysis for CO_2_ removal [56] or wet pretreatment methods such as mineral/organic acid or ammonium salt leaching [57]. However, such a pretreatment method applied before the thermochemical activation of slags would cause further problems, such as the partial leaching of Cr (and V), the need for further waste liquor management, and higher expenses. While calcium carbonate removal would benefit the subsequent thermochemical process, the economic viability of the above-mentioned pretreatment methods for CaCO_3_ or CO_2_ removal from slag requires further study.

Figure 16b indicates that V extraction exhibits different dynamics compared to Cr, which is related to its higher reactivity and phase distribution within the slag. In the case of the EAF slag CH1, the V yield at 500 °C remains within a more stable range, with a maximum extraction of 49.7% achieved at a dosage of 8 g NaOH, corresponding to the optimum for Cr. However, unlike Cr, V does not show such a dramatic decrease in yield in the presence of excess reagent (12 g).

In contrast, a generally more favorable effect of higher NaOH dosages was observed for the CH2 slag. The highest Cr yield (80.6%) was achieved using 16 g of NaOH, which indicates a different reaction behavior of the CH2 slag, likely related to its low Fe content (0.4% Fe, Table 1). The beneficial effect of excess NaOH observed for the CH2 slag lies in the lower consumption of the reagent in reactions with iron phases; consequently, the excess NaOH improves melt fluidity and mass transport by homogeneously distributing the molten phase throughout the slag sample, thus facilitating better reactions in the liquid phase and subsequent extraction process by making the reagent more accessible in the system.

For the CH2 slag, the V extraction process is smoother and less dependent on increasing the NaOH dosage above 8 g, with the achieved yields reaching a plateau at approximately 42%. The lower V yield from the FeCr slag compared to the EAF steelmaking slag indicates that it is locked more tightly within the silicate matrix, which only partially decomposes at 500 °C.

From the overall comparison, it can be concluded that while the NaOH dosage is critical for maximizing Cr extraction from CH1, V extraction exhibits a higher tolerance to varying activation conditions. The efficiency of the thermal conversion process in CH1 slag is determined by the balance of the iron, calcium and sodium content.

#### 3.2.3. Analysis of Thermochemically Activated Slags with NaOH and Leaching Residues

##### Analysis of Thermochemically Activated Slag CH1 and Leaching Residues

The content of the monitored elements in the CH1 slag—thermochemically activated under the conditions of 12 g of CH1, 8 g of NaOH, 500 °C, and 2 h—as well as the concentration of these elements in the leachate obtained by water leaching of the activated CH1 slag (L:S = 20, T = 60 °C, 5 min), is presented in Table 5.

**Figure 19:**
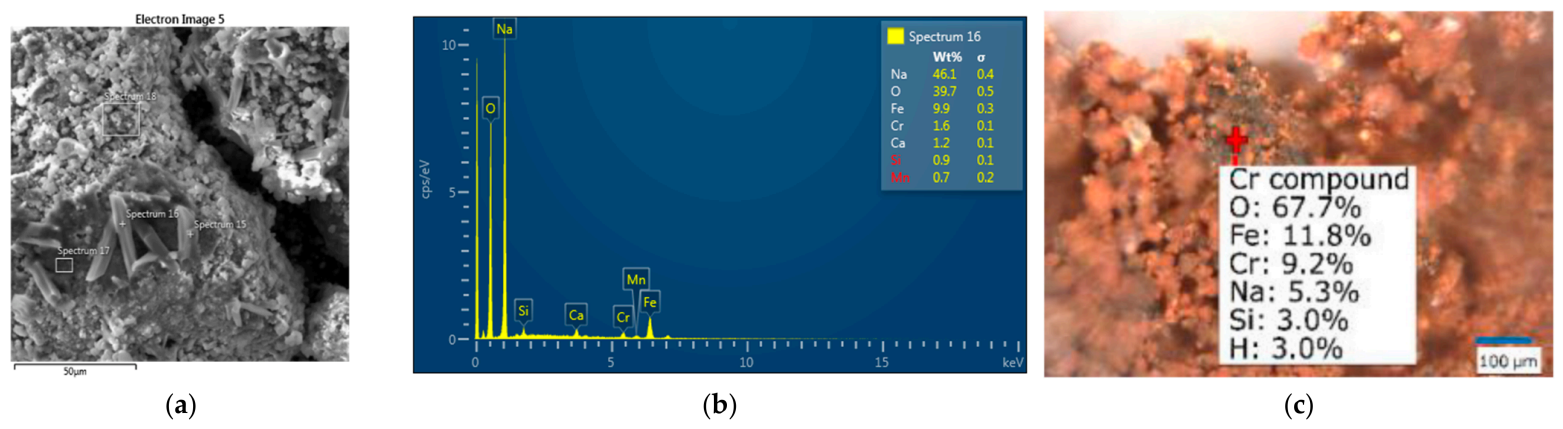
Thermochemically activated CH1 sample (500 °C, CH1:NaOH = 12:8 g, 2 h): (**a**) SEM micrograph of the bulk material morphology; (**b**) EDS spectrum of point 16 rod-like particles of NaFeO_2_ with Cr; (**c**) optical micrograph with LIBS point analysis of grey particles within the activated slag (Na-Fe-Cr oxides/hydroxides).

The morphological characterization and elemental composition of the activated CH1 slag, verified via SEM/EDS and optical microscopy with LIBS analysis, are documented in Figure 19.

XRD analysis of the activated CH1 slag (Figure 17) reveals that various Na-based phases with varying degrees of solubility (Na_2_MgSiO_4_, Na_2_CaSiO_4_) were formed during the thermochemical activation process at 500 °C. The presence of these phases is consistent with findings reported in the literature [55,58]. Further observation of the XRD pattern confirmed that the predominant phase in the activated CH1 slag is NaFeO_2_, which aligns directly with the high Fe content (31.82%) of the original carbon steel EAF slag and thermodynamic predictions (reactions (33) and (34)). This was also indicated by the SEM micrograph in Figure 19a. α-NaFeO_2_ often appears as agglomerated, hexagonal, columnar particles, depending on the thermal treatment conditions [59].

Although XRD analysis (Figure 17, red pattern) could not identify Na-based forms of Cr in the CH1 activated sample with NaOH at 500 °C, the LIBS (Figure 18b) and SEM-EDS analyses (Figure 19b) revealed the presence of Na_2_CrO_4_ and Cr within the NaFeO_2_ particles, respectively. Similarly, the LIBS analysis of the CH1 sample (Figure 19c) indicates Cr in association with Fe and Na oxide (grey particles). This is consistent with the SEM-EDS analysis and thermodynamic study, according to reactions (1) and (2), in which Na_2_CrO_4_ and Fe_2_O_3_ are formed during the activation of slag. Alternatively, it represents a complex Na-Fe-Cr oxide.

Based on the findings of this study, it is evident that the high Fe content of the CH1 slag, together with the presence of CaCO_3_, poses a problem in the alkaline thermochemical treatment of slag. The Fe and Ca phases react and consume a large proportion of the alkaline reagent. This has a negative effect on the availability of NaOH for reactions with specific Cr-bearing phases. On the other hand, chemical analyses confirmed that Cr and V concentrations in the solution remained stable, proving that no undesirable absorption (occlusion) in the precipitate or adsorption onto the surface of the freshly formed Fe hydroxides occurred.

The analyses also reveal that Cr is unevenly distributed within the compounds of the CH1 slag, suggesting a possible insufficient amount of NaOH available for complete stoichiometric transformation of the Cr-containing refractory phases. Furthermore, an assessment of the chemical affinities of various Fe-bearing oxides towards NaOH reveals a distinct hierarchical order. FeO exhibits the highest reactivity—governed by reaction (33)—followed by the complex spinel FeCr_2_O_4_ (reaction (2)), while Fe_3_O_4_ (reaction (34)) demonstrates the lowest relative affinity. This observed trend is further corroborated by the XRD pattern in Figure 17, where the transformation of FeO was nearly complete (negligible FeO remaining), while a substantial amount of the Fe_3_O_4_ phase remained in the activated sample. Furthermore, as evidenced by XRD analysis (Figure 17), Na_2_CO_3_ was identified in the activated CH1 sample. This phase is formed “in situ” during the activation process according to reactions (37) and (38). Unlike NaOH, Na_2_CO_3_ is less reactive toward Fe oxides; specifically, the thermodynamic probability for NaFeO_2_ formation via Na_2_CO_3_ (40) is negligible compared to the efficient activation pathways provided by NaOH (33) and (34).

*4Fe*_3_*O*_4_* + 6Na*_2_*CO*_3_* + O*_2*(g)*_* = 12NaFeO*_2_* + 6CO*_2*(g)*_*, ΔG*^0^_773.15_* = 10.56* kJ (40)

Thus, because Na_2_CO_3_ remains solid at 500 °C (T_melt_ = 851 °C [60]), its formation suppresses the liquid phase required for efficient reactions and metal extraction. This phenomenon represents an important factor behind the lower Cr/V leaching efficiency from the activated CH1 samples.

**Figure 20:**
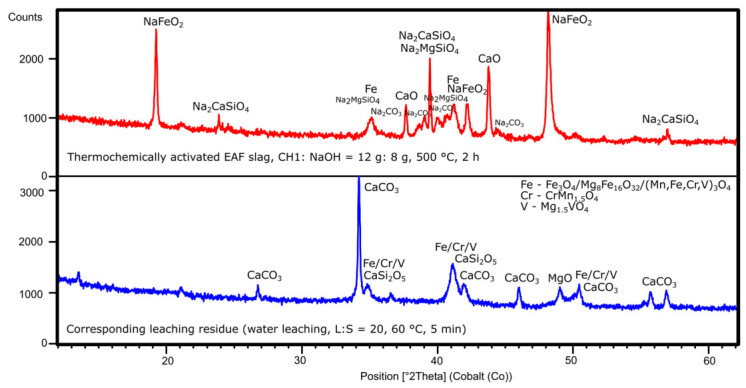
Comparison of XRD diffractograms of the activated CH1 slag at 500 °C and the corresponding CH1 leaching residue.

The selectivity and efficiency of the water leaching process are further evidenced by the phase evolution between the activated CH1 slag and the final leaching residue (Figure 20).

XRD analysis of the leaching residue (Figure 20, blue pattern) confirmed changes in the phase composition, consistent with a weight loss of approximately 30 wt.% in the solid fraction. This corresponds to the effective removal of water-soluble Na compounds formed during thermochemical activation (Figure 20, red pattern)—such as chromates, vanadates, and soluble silicates—as well as the partial removal of Fe due to the hydrolysis of NaFeO_2_. The leaching residue is primarily composed of a newly formed insoluble CaCO_3_ and persistent spinel-type phases. While the absence of Mg in the leachate suggests its potential incorporation into secondary phases (e.g., hydrotalcite-like structures) [61], the XRD pattern is dominated by a stable crystalline matrix containing complex Mg-Fe spinels and Cr/V-bearing oxides (specifically CrMn_1.5_O_4_ and Mg_1.5_VO_4_). These phases represent unreacted components that failed to interact with the NaOH reagent, directly accounting for the observed limitations in Cr and V recovery.

**Figure 21:**
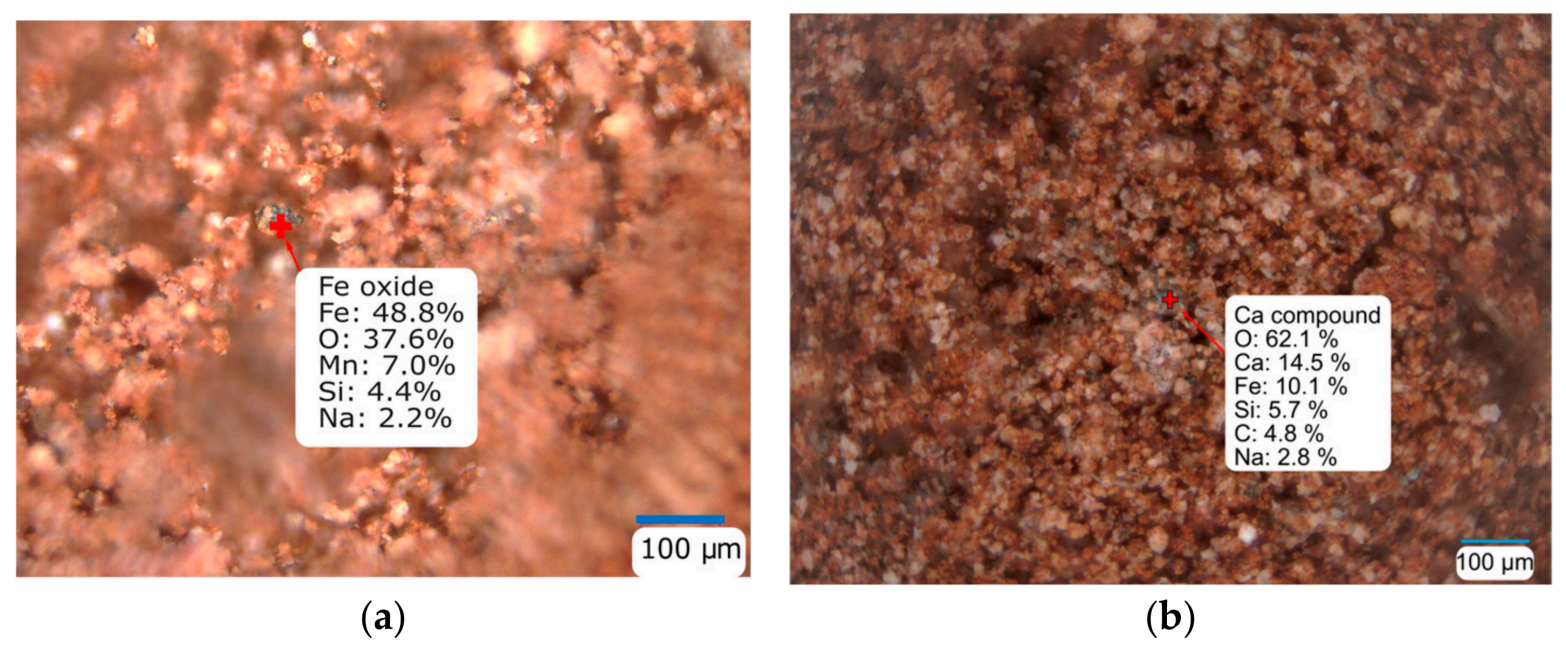
LIBS analysis of leaching residue of CH1: (**a**) unreacted Fe-Mn oxide, (**b**) CaCO_3_ particle with iron species.

These findings are further supported by microscopic observations (see Figure 21a,b), which reveal the morphology of the leaching residue of activated slag CH1. Furthermore, the optical micrograph, combined with LIBS point analysis and XRD analysis (Figure 20), displays and confirms the presence of individual, unreacted Fe-Mn oxides and indicates newly formed CaCO_3_ particles embedded within the porous matrix.

##### Analysis of CH2 Thermochemically Activated Slag and Leaching Residues

Table 6 presents the elemental composition of the thermochemically activated CH2 slag (obtained under the conditions: 12 g CH2, 8 g NaOH, 500 °C, 2 h) and the concentrations of these elements in the leachate produced by water leaching of the thermochemically activated CH2 slag (L:S = 20, 60 °C, 5 min).

**Figure 22:**
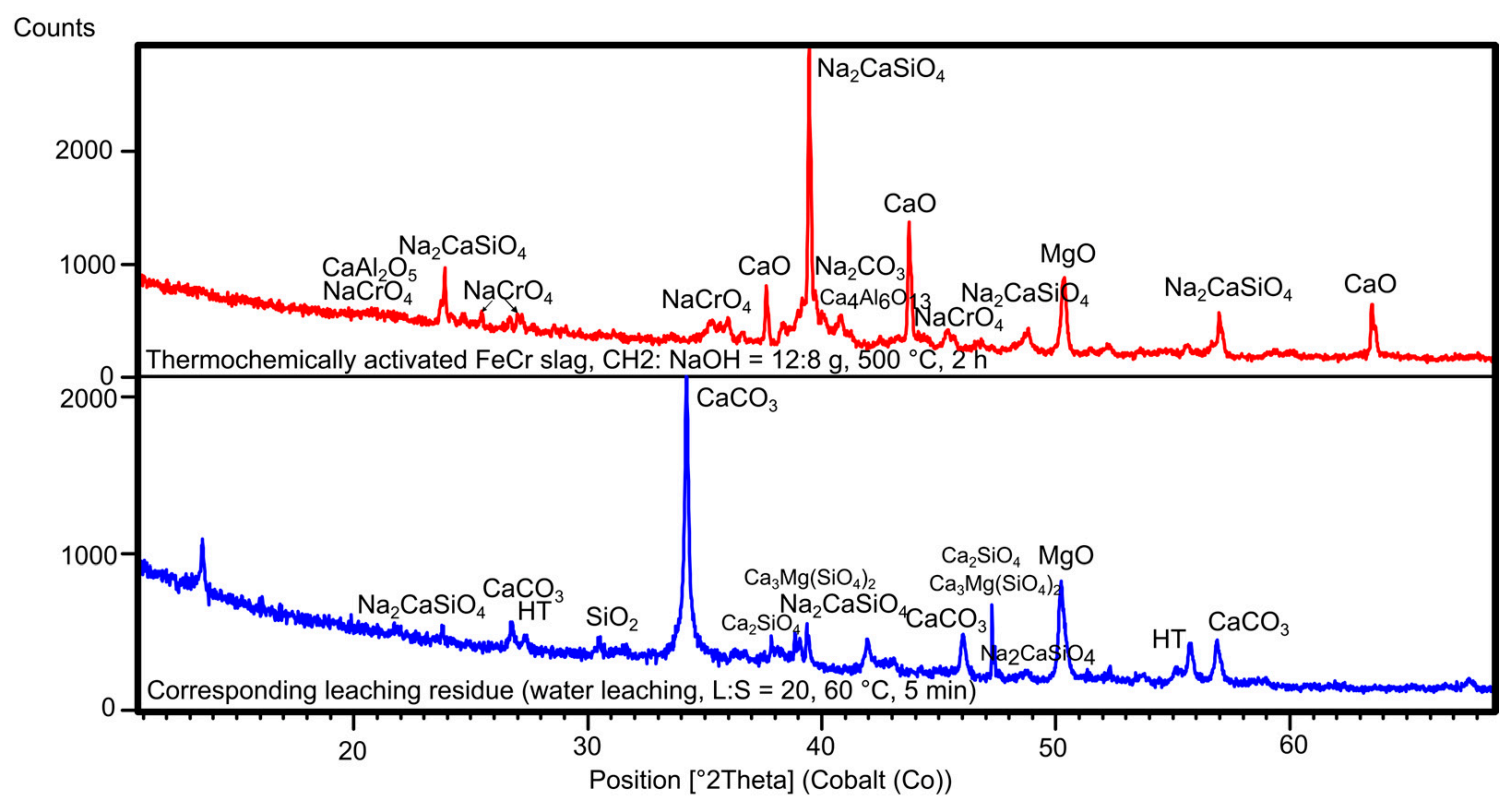
Comparison of XRD diffractograms of the activated CH2 slag at 500 °C and the corresponding CH2 leaching residue.

Figure 22 presents a comparison of the XRD diffractograms of the activated CH2 slag and its corresponding leaching residue.

The XRD diffractogram of the activated CH2 sample (Figure 22, red pattern) confirmed Na_2_CaSiO_4_ as the major phase. The formation of this phase, along with CaO and MgO, indicates the decomposition of the primary silicate minerals, such as merwinite merwinite (Ca_3_MgSi_2_O_8_) and bredigite (Ca_14_Mg_2_Si_8_O_32_), during the thermochemical activation process. This transformation is critical; the resulting Na_2_CaSiO_4_ phase is readily water-soluble, and its dissolution creates a porous architecture within the slag matrix. This increased porosity facilitates the effective penetration of the released NaOH reagent deep into the grain core, which subsequently promotes the decomposition of the refractory spinel phases (FeCr_2_O_4_, MgCr_2_O_4_) where Cr is predominantly bound [55]. The identification of the soluble Na_2_CrO_4_ phase in the activated sample is in alignment with thermodynamic studies (reaction (2)). However, the thermodynamically predicted soluble vanadium-based phases were not confirmed by XRD analysis in the activated CH2 sample at 500 °C.

Furthermore, the presence of various calcium aluminates (e.g., CaAl_2_O_5_, Ca_4_Al_6_O_13_) confirms the complex mutual reactions between the slag components and the reagent. The initial Ca-bearing phases identified in the CH2 slag (Figure 7) reacted with NaOH during the thermochemical activation, leading to the formation of new mineralogical species.

XRD analysis of the leaching residue following the processing of the CH2 slag (Figure 22, blue pattern) confirmed the absence of the Na_2_CrO_4_ phase, demonstrating that most of the Cr was transformed into a soluble form during thermochemical activation and subsequently leached. The analysis of the residue confirmed the presence of CaCO_3_, Na_2_CaSiO_4_, Ca_2_SiO_4_, Ca_3_Mg(SiO_4_)_2_, MgO, SiO_2_, and hydrotalcite-like phases with the chemical formula ((Mg_4_Al_2_)(OH)_12_CO_3_(H_2_O)_3_)_0.5_. The absence of Na_2_CO_3_ in the leaching residue compared to the activated sample indicates its effective dissolution. The continued presence of Na_2_CaSiO_4_ and other silicates in the residue indicates limited solubility under the given conditions, likely requiring a higher L:S ratio or an extended leaching time. A weight loss of approximately 20 wt.% following the leaching suggests that while some Na compounds are successfully removed, the matrix of the thermally activated CH2 slag remains relatively stable, as evidenced by the persistence of these crystalline silicate phases. The low silicon concentration in the CH2 leachate (42 µg/mL) compared to its high content in the activated sample confirms the chemical stability of the silicate matrix. The bulk of the silicon remains fixed in the solid residue, consistent with the XRD findings, which highlight the presence of these stable, low-solubility silicate species.

### 3.3. Synthesis of Results and Proposal for a Combined Recycling Process for Cr- and V-Bearing Slags

Based on the screening of a wide range of thermochemical activation reagents and the subsequent detailed study of process parameters using the NaOH reference system, it can be concluded that low-temperature thermochemical activation is an effective method for breaking down spinel and silicate structures in carbon steel EAF and FeCr slags.

Experiments confirmed that temperatures within the 300–500 °C range lead to the transformation of resistant Cr and V phases into soluble forms. Increasing the temperature to 600 °C did not yield a rise in Cr recovery, which defines 500 °C as the upper limit of process efficiency under the given conditions. Conversely, temperatures below 300 °C are insufficient to disrupt the stable spinel structure of the slags.

The selection of the optimal thermochemical activation reagent depends on the target metal and the specific matrix type:

Vanadium Recovery (carbon steel EAF slag; CH1): To optimize V recovery, the use of KOH at 400 °C is most effective, achieving a yield of up to 89% at a low slag-to-KOH ratio of 4/16.

Vanadium Recovery (FeCr slag; CH2): Under the same conditions (KOH, 400 °C), V recovery from CH2 reached 54.5%.

Chromium Recovery (carbon steel EAF slag; CH1): To achieve the highest Cr recovery using NaOH, a ratio of 8 g NaOH/12 g slag at 500 °C proved most efficient, with a maximum yield of nearly 62% Cr.

Chromium Recovery (FeCr slag; CH2): To maximize Cr recovery from the CH2 slag using NaOH, a ratio of 16 g NaOH/12 g slag at 500 °C proved most effective, achieving a maximum Cr yield of nearly 81%.

Combined Cr and V Extraction (carbon steel EAF slag; CH1): For the simultaneous extraction of both metals, a NaOH–NaCl mixture at 500 °C is recommended, ensuring Cr and V recoveries in the range of 56–57%.

The maximum concentration of metals in the leachate is reached after only 5 min of water leaching at 60 °C, indicating the excellent solubility of the newly formed phases. The addition of oxidizing agents (Na_2_O_2_, NaNO_3_) during thermochemical activation did not yield the expected increase in recovery. This suggests that molten NaOH alone, in the presence of atmospheric oxygen, provides sufficient oxidative potential for the conversion of Cr^3+^ to soluble Cr^6+^.

A technological advantage of the procedure studied is its high selectivity. Although Fe (particularly in the carbon steel EAF slag) largely transforms into soluble NaFeO_2_, it subsequently precipitates as insoluble oxides/hydroxides through spontaneous hydrolysis during leaching and cooling of the leachate. This process occurs without the undesirable coprecipitation of the target metals (Cr and V). However, a certain technological barrier may be posed by the filtration of fine Fe precipitates, as well as the presence of residual magnetite/ferrites and other resistant phases in the activated samples, which limit overall metal recovery. While Cr extraction ranges from approximately 60% (CH1) to 80% (CH2), the incomplete leaching of Cr and V points to several potential directions for achieving higher metal yields. These include:

Optimization of thermochemical activation duration, rather than further increasing the NaOH content or thermal activation temperature, to ensure the thorough decomposition of stable Cr and V phases.

Leaching can be further intensified because the current limiting factors are likely the L:S ratio and the system’s high pulp density and viscosity. Leaching efficiency could be further enhanced by incorporating ultrasonic or microwave irradiation, as well as mechanochemical activation (e.g., high-energy milling of the activated slag). Such methods would facilitate the more efficient release and diffusion of sodium-based Cr and V species through the slag matrix.

These considerations regarding efficiency and selectivity are further differentiated by the unique behavior of the CH2 matrix. The limited decomposition of the slag matrix in the FeCr slag (CH2) fundamentally contributes to the process selectivity, enabling the effective leaching of target metals (Cr, V) while maintaining high leachate purity. A comparison of the chemical compositions of the CH1 and CH2 activated samples at 500 °C and their respective leachates (Table 5 and Table 6) reveals that the matrix of the thermally activated CH2 slag exhibits higher structural stability and chemical resistance compared to that of CH1. Specifically, despite CH1 containing only 6% Si, its leachate had the same Si concentration as that of the CH2 sample, which had a three-fold higher Si content. This difference is likely related to the notably lower Fe content in CH2; while in the CH1 slag, Fe acts as a flux promoting phase transformations of the matrix, the CH2 structure remains more robust.

A significant finding is the identification of a secondary hydrotalcite-type phase exclusively in the leaching residue of the thermally activated CH2 slag. The presence of the original SiO_2_ and Ca_2_SiO_4_ phases confirms that a portion of the silicate matrix remained intact after the process. The formation of hydrotalcite effectively immobilizes Mg, which explains the negligible concentration of Mg in the leachate of the CH2 slag. Hydrotalcite (HT) is an inherently stable phase that fixes structural components like Mg and Al within its layers; furthermore, it is able to trap hazardous species, effectively preventing the remobilization of contaminants into the environment [62]. This mechanism, alongside the presence of stable CaCO_3_, suggests that the CH2 leaching residue maintains excellent structural integrity, making it a promising candidate for further industrial applications, such as a sorbent.

Regarding vanadium, it was not explicitly detected in the XRD analysis of either the CH1 or CH2 activated samples. This is likely attributed to its low concentration in the slag and/or its presence in the form of highly dispersed phases or isomorphous substitutions within the spinel or silicate lattices. Nevertheless, leaching experiments demonstrated its significant extraction, confirming that V is available for selective recovery despite the limitations in its phase identification.

Building upon these observations, the overall process selectivity is further reinforced by the specific behavior of the accompanying matrix elements during thermochemical activation and subsequent leaching:

Calcium: In the initial samples, calcium is primarily present as CaCO_3_ and silicates. During thermochemical activation with NaOH, CaCO_3_ reacts to form CaO and water-soluble Na_2_CO_3_. The identification of Na_2_CaSiO_4_ phases in the activated samples of both slags indicates that Ca actively binds to silicon during thermal activation. This prevents the formation of viscous sodium silicates and facilitates the penetration of the reagent to the spinels. During subsequent leaching, Ca is absent from the solution due to back-carbonation and the formation of stable calcite (CaCO_3_) in the leaching residue.

Silicon: Although partial decomposition of initial silicate phases (Na_2_CaSiO_4_) occurs, the silicon concentration in the solution remains low (approx. 42–48 µg/mL). This Si stability, consistent across both slag types, confirms the chemical robustness of the silicate matrix under the specified leaching conditions. The bulk of the silicon remains sequestered in the solid residue, predominantly as stable crystalline phases that resist further leaching. Specifically, in the CH1 slag, these are associated with persistent spinel-type structures and an unreacted silicate matrix, whereas in the CH2 slag, the residue is characterized by the persistence of highly stable crystalline silicates (e.g., Ca_2_SiO_4_, Ca_3_Mg(SiO_4_)_2_).

Magnesium is essentially absent from the leachate of both slag types. In the CH2 slag, this immobilization was explicitly confirmed by XRD analysis through the identification of a secondary hydrotalcite phase—a complex hydrated magnesium–aluminium carbonate. While this mechanism is most pronounced in CH2, the formation of similar Mg-compounds is possible for Mg fixation in the CH1 slag as well.

The synergy of Ca, Si, Mg, and Fe immobilization within the leaching residue facilitates the production of a high-purity Cr and V solution while ensuring the long-term chemical stability of the mineral matrix. The formation of secondary HT, particularly in the insoluble residue of the CH2 slag, combined with calcite, provides the leaching residue with specific material and surface properties.

Theoretically, this residue could represent a promising adsorbent for contaminant capture, thereby contributing to a closed-loop slag processing cycle. Furthermore, it can be assumed that the stabilized matrix could, after potential further treatment, meet environmental criteria for construction applications. To confirm these assumptions and establish the limits for its safe utilization, further detailed research is essential, focusing particularly on the long-term stability of the fixed elements, specific surface area (SSA) and the testing of sorption capacities.

Beyond the stabilization and utilization of the mineral residual matrix, the resulting leachate presents a high potential for the recovery of commercially valuable products, opening several research avenues:

- Chromium Recovery: Selective precipitation in the form of chromates (e.g., BaCrO_4_ or CaCrO_4_), which are widely used in the pigment industry. However, the purity of the final pigment could be affected by residual matrix elements (specifically Al, Si, Fe and Ca) that are incorporated into the precipitate during the process. These impurities can induce lattice defects or form secondary phases, which negatively impact the pigment’s color purity, crystallinity, and chemical stability. Therefore, the leachate must be refined prior to chromium precipitation to ensure the production of high-quality pigments.
- Vanadium Recovery: Isolation in the form of polyvanadates or ammonium metavanadate (NH_4_VO_3_). These are key precursors in the production of ferroalloys or modern V redox flow batteries.

**Figure 23:**
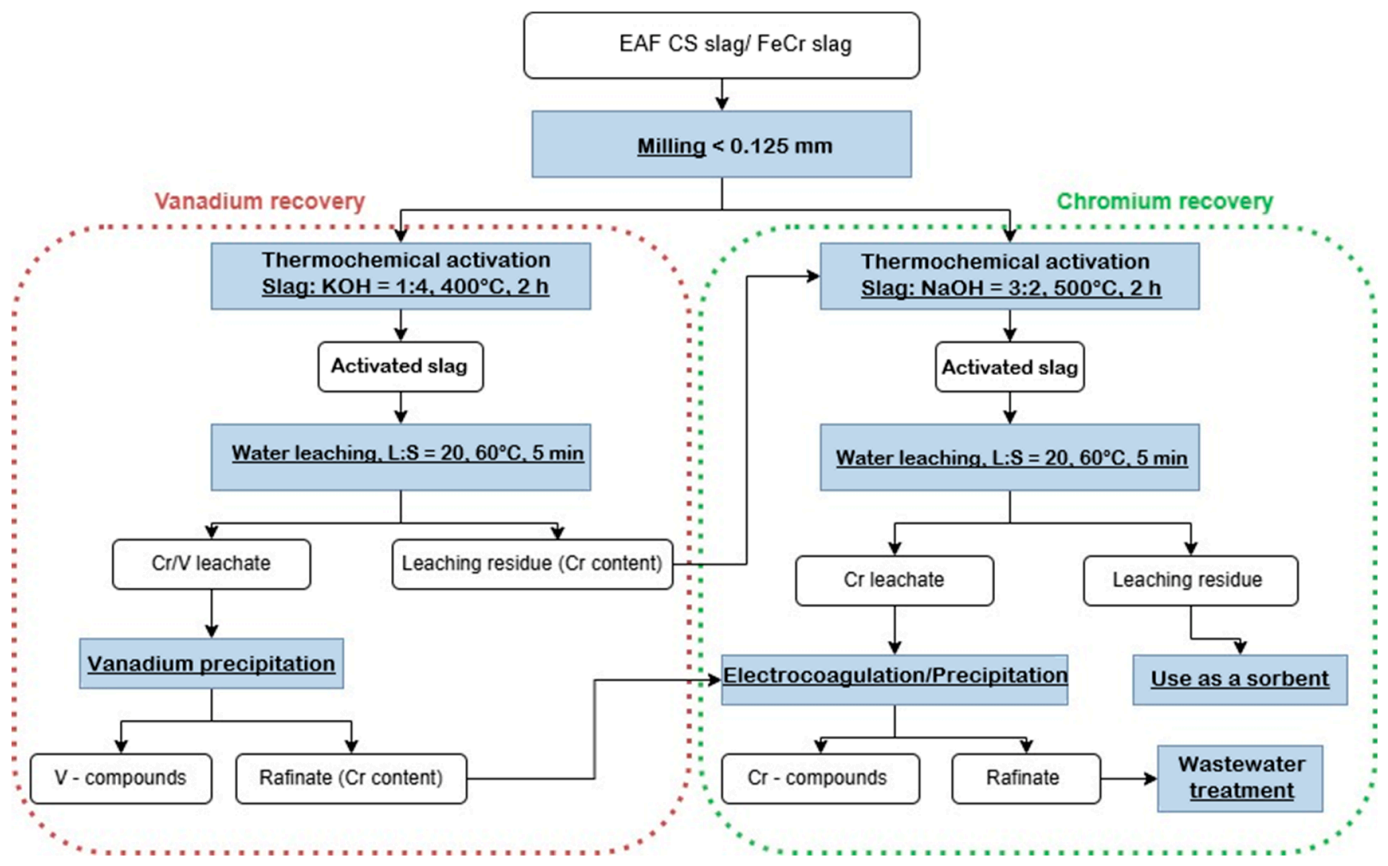
Proposed recycling process for Cr- and V-bearing slags using thermochemical activation.

The integrated analysis of the experimental parameters and thermodynamic feasibility has led to the development of a conceptual recycling flowsheet for Cr- and V-containing slags, as illustrated in Figure 23. This flowsheet outlines the strategic processing steps required for effective metal recovery and byproduct valorization. While a comprehensive techno-economic assessment is beyond the scope of this study, the economic viability of the process is primarily driven by the valorization of the slag. By stabilizing the mineral matrix and recovering critical raw materials, the process transforms waste containing hazardous substances into secondary raw material suitable, e.g., for the construction sector. This approach not only generates value from recovered strategic metals but also avoids the considerable long-term costs and environmental liabilities associated with the landfilling of untreated slag, representing a significant driver for the implementation of such circular economy strategies in industrial practice.

## 4. Conclusions

The presented scientific study confirms that low-temperature thermochemical activation represents a promising and energy-efficient alternative to traditional methods for processing carbon steel EAF and FeCr slags. This work provides a comprehensive insight into the decomposition mechanisms of resistant spinel and silicate structures, which constitute a technological barrier to slag recycling. Experimental results demonstrate that through the appropriate selection of process parameters, high extraction selectivity of target metals (Cr, V) can be achieved while simultaneously stabilizing the slag matrix.

The key findings of this work include:

- Process Sensitivity: It was experimentally confirmed that the process is highly sensitive to temperature and the type of reagent used. The highest V extraction rate (89.4% for sample CH1) was achieved at 400 °C using KOH. For the combined extraction of Cr and V, a NaOH–NaCl eutectic mixture at 500 °C appears to be the optimal medium, ensuring stable recovery of both metals above 55%.
- Self-Purifying Mechanism: A significant finding is the system’s ability to purify the alkaline leachate “in situ” during or after leaching. Iron, following its initial conversion to NaFeO_2_ in the activated sample, undergoes immediate hydrolysis into stable oxides/hydroxides in the strongly alkaline environment (pH 12–13.6). The synergy with the immobilization of Ca and Mg (Al) into secondary mineral phases (calcite and hydrotalcite) enables the production of high-purity solutions with minimal contamination from matrix elements.

To advance this technology towards industrial application, further efforts must focus on optimizing thermochemical activation parameters (e.g., thermal activation duration) to ensure the complete decomposition of residual spinels and stable silicates. Subsequent steps should address the intensification of leaching (L:S ratio, mechanochemical activation of the slag/activated sample or the use of ultrasound). An equally important task will be developing procedures to process the resulting leachates into commercial products, such as pigments and battery precursors, as well as studying the applicability of the stabilized residue and characterizing its sorption properties.

## Figures and Tables

**Table 1:** Chemical composition of slag samples CH1 and CH2 (determined by HR-CS FAAS).

Sample	Content [wt.%]							
	Cr	V	Fe	Mg	Ca	Al	Si	Residue
CH1	2.85 ± 0.06	0.0717 ± 0.0015	31.82 ± 0.65	1.40 ± 0.03	17.37 ± 0.35	4.24 ± 0.09	4.54 ± 0.10	37.71 ± 0.8
CH2	3.64 ± 0.07	0.016 ± 0.0004	0.428 ± 0.010	3.89 ± 0.08	31.90 ± 0.65	2.29 ± 0.05	13.49 ± 0.30	44.35 ± 0.9

**Table 2:** ΔG^0^ for selected chemical reactions of potential slag components during oxidative thermochemical activation at 200–800 °C.

No.	Chemical Reaction	T [°C]		
		200	400	800
		ΔG^0^ [kJ]		
(1)	*Cr_2_O_3_ + **4NaOH** + 1.5O_2(g)_ = 2Na_2_CrO_4_ + 2H_2_O_(g)_*	*−364.1*	*−355.7*	*−326.0*
(2)	*4FeCr_2_O_4_ + **16NaOH** + 7O_2(g)_ = 8Na_2_CrO_4_ + 2Fe_2_O_3_ + 8H_2_O_(g)_*	*−590.7*	*−556.6*	*−478.6*
(3)	*Mg_2_V_2_O_7_ + **2NaOH** = 2NaVO_3_ + 2MgO + H_2_O_(g)_*	*−110.4*	*−125.0*	*−148.1*
(4)	*Mg_2_V_2_O_7_ + **6NaOH** = 2Na_3_VO_4_ + 2MgO + 3H_2_O_(g)_*	*−245.5*	*−287.2*	*−315.0*
(5)	*Mg_2_V_2_O_7_ + **3Na_2_O_2_** = 2Na_3_VO_4_ + 2MgO + 1.5O_2(g)_*	*−468.6*	*−514.9*	*−582.4*
(6)	*Mg_2_V_2_O_7_ + **2Na_2_O_2_** + **2NaOH** = 2Na_3_VO_4_ + 2MgO + H_2_O_(g)_ + O_2(g)_*	*−394.2*	*−439.0*	*−493.3*
(7)	*Mg_3_(VO_4_)_2_ + **6NaOH** = 2Na_3_VO_4_ + 3MgO + 3H_2_O_(g)_*	*−232.7*	*−282.7*	*−340.3*
(8)	*Mg_3_(VO_4_)_2_ + **2NaOH** = 2NaVO_3_ + 3MgO + H_2_O_(g)_*	*−97.6*	*−120.6*	*−173.3*
(9)	*Mg_3_(VO_4_)_2_ + **3Na_2_O_2_** = 2Na_3_VO_4_ + 3MgO + 1.5O_2(g)_*	*−455.8*	*−510.5*	*−607.6*
(10)	*Mg_3_(VO_4_)_2_ + **2Na_2_O_2_** = Na_4_V_2_O_7_ + 3MgO + O_2(g)_*	*−336.4*	*−379.8*	*−474.4*
(11)	*2 Mg_3_(VO_4_)_2_ + **2Na_2_O_2_** = 4NaVO_3_ + 6MgO + O_2(g)_*	*−344.0*	*−393.0*	*−524.8*
(12)	*MgCr_2_O_4_ + 1.5O_2(g)_ + **4NaOH** = 2Na_2_CrO_4_ + MgO + 2H_2_O_(g)_*	*−315.3*	*−311.7*	*−298.8*
(13)	*2Cr_2_O_3_ + **4Na_2_O_2_** + O_2(g)_ = 2Na_2_Cr_2_O_7_ + 2Na_2_O*	*−424.1*	*−350.0*	*−89.3*
(14)	*2Cr_2_O_3_ + **3Na_2_O_2_** = 2Na_2_CrO_4_ + Na_2_O*	*−453.6*	*−464.1*	*−485.8*
(15)	*4FeCr_2_O_4_ + **12Na_2_O_2_** + O_2(g)_ = 8Na_2_CrO_4_ + 2Fe_2_O_3_ + 4Na_2_O*	*−2267.9*	*−2258.0*	*−2248.6*
(16)	*4FeCr_2_O_4_ + **14Na_2_O_2_** = 8Na_2_CrO_4_ + 2Fe_2_O_3_ + 6Na_2_O*	*−1969.7*	*−1994.9*	*−2046.5*
(17)	*2Mg_2_VO_4_ + **2Na_2_O_2_** = 4NaVO_3_ + 4MgO + O_2(g)_*	*−560.7*	*−601.4*	*−676.1*
(18)	*2MgCr_2_O_4_ + **4Na_2_O_2_** + O_2(g)_ = 4Na_2_CrO_4_ + 2MgO*	*−928.2*	*−926.9*	*−953.9*
(19)	*MgCr_2_O_4_ + **3Na_2_O_2_** = 2Na_2_CrO_4_ + MgO + Na_2_O*	*−404.9*	*−420.0*	*−458.6*
(20)	*Cr_2_O_3_ + **3Na_2_O_2_** + **2NaOH** = 2Na_2_CrO_4_ + 2Na_2_O + H_2_O_(g)_*	*−320.1*	*−344.7*	*−378.4*
(21)	*FeCr_2_O_4_ + **3.5Na_2_O_2_** + **3NaOH** = 2Na_2_CrO_4_ + Fe(OH)_3_ + 3Na_2_O*	*−246.2*	*−240.3*	*−207.9*
(22)	*MgCr_2_O_4_ + **3Na_2_O_2_** + **2NaOH** = 2Na_2_CrO_4_ + MgO + 2Na_2_O + H_2_O_(g)_*	*−271.3*	*−300.7*	*−351.2*
(23)	*Cr_2_O_3_ + **4KOH** + 1.5O_2(g)_ = 2K_2_CrO_4_ + 2H_2_O_(g)_*	*−485.0*	*−481.5*	*−445.5*
(24)	*2FeCr_2_O_4_ + **8KOH** + 3.5O_2(g)_ = 4K_2_CrO_4_ + Fe_2_O_3_ + 4H_2_O_(g)_*	*−1106.8*	*−1075.7*	*−964.0*
(25)	*Mg_2_V_2_O_7_ + **4KOH** = K_4_V_2_O_7_ + 2MgO + 2H_2_O_(g)_*	*−202.3*	*−196.8*	*−92.9*
(26)	*Mg_2_V_2_O_7_ + **6KOH** = 2K_3_VO_4_ + 2MgO + 3H_2_O_(g)_*	*−246.2*	*−246.4*	*−121.9*
(27)	*Mg_3_(VO_4_)_2_ + **6KOH** = 2K_3_VO_4_ + 3MgO + 3H_2_O_(g)_*	*−236.7*	*−244.9*	*−149.8*
(28)	*Mg_3_(VO_4_)_2_ + **4KOH** = K_4_VO_7_ + 3MgO + 2H_2_O_(g)_*	*−192.8*	*−195.3*	*−120.8*
(29)	*MgCr_2_O_4_ + **4KOH** + 1.5O_2(g)_ = 2K_2_CrO_4_ + MgO + 2H_2_O_(g)_*	*−439.5*	*−437.9*	*−407.5*
(30)	*MgCr_2_O_4_ + **2KOH** + 1.5O_2(g)_ = K_2_Cr_2_O_7_ + MgO + H_2_O_(g)_*	*−239.2*	*−222.4*	*−204.9*

**Table 3:** Experimental conditions for thermochemical activation of CH1 and CH2 slags and subsequent water leaching (Leaching conditions: 10 g slag sample, 200 mL H_2_O, L:S = 20, T = 60 °C, 500 rpm, t = 1 h).

Experimental Set	Thermochemical Activation Conditions for CH1 and CH2 Followed by Water Leaching
1.	12 g slag, **8 g NaOH,** 400 °C, 2 h
2.	12 g slag, **8 g NaOH, 0.8 g NaNO_3_**, 500 °C, 2 h
3.	12 g slag, **8 g NaOH**, **4 g NaCl**, 500 °C, 2 h
4.	15 g slag**, 15 g NaOH, 6 g Na_2_O**_2_, 500 °C, 3 h
5.	10 g slag, **4 g KOH, 6 g K_2_CO_3_**, 400 °C,2 h
6.	4 g slag**, 16 g KOH**, **400 °C**, 2 h
7.	12 g slag, **12 g Na_2_O_2_**, **500 °C**, 2 h
8.	3 g slag, **12 g (NH_4_)_2_SO_4_,** 1 g **KHSO_4_,** 350 °C, 0.5 h

**Table 4:** Experimental conditions for CH2 slag thermochemical activation followed by acidic leaching (leaching conditions: 10 g of activated slag, 400 mL 3 M H_2_SO_4_, L:S = 40, T = 60 °C, 300 rpm, t = 1 h).

Experimental Set	Thermochemical Activation Conditions for CH2 Followed by Leaching in 3M H_2_SO_4_
9.	12 g slag, **15 g K_2_S_2_O_5_**, 500 °C, 2 h
10.	12 g slag, **15 g K_2_S_2_O_7_**, 500 °C, 2 h
11.	12 g slag, **12 g Na_2_SO_4_ ·10H_2_O**, 300 °C, 2 h

**Table 5:** Elemental composition of activated CH1 slag (12 g CH1, 8 g NaOH, 500 °C, 2 h) and the relevant leachate.

Sample	Units	Monitored Elements						
		Cr	V	Fe	Mg	Ca	Al	Si
Thermochemically activated CH1	[wt. %]	1.8 ± 0.05	0.039 ± 0.001	17.33 ± 0.72	0.749 ± 0.03	13.08 ± 0.40	1.83 0.05	6.29 ± 0.19
Leachate	[µg/mL]	514.6 ± 15.4	8.83 ± 0.3	BDL *	BDL	BDL	412.0 ± 17	48.16 ± 1.44

* BDL—Below Detection Limit.

**Table 6:** Elemental composition of the activated CH2 slag (12 g CH2, 8 g NaOH, 500 °C, 2 h) and the relevant leachate.

Sample	Units	Monitored Elements						
		Cr	V	Fe	Mg	Ca	Al	Si
Thermochemically activated CH2	[wt. %]	2.51 ± 0.09	0.017 ± 0.001	0.27 ± 0.01	2.31 ± 0.07	20.91 ± 0.98	1.37 ± 0.05	15.38 ± 0.66
Leachate	[µg/mL]	680.1 ± 30.4	3.673 ± 0.2	3.03 ± 0.12	BDL *	BDL	274.4 ± 10.2	42.33 ± 1.67

* BDL—Below Detection Limit.

## Data Availability

The original contributions presented in this study are included in the article. Further inquiries can be directed to the corresponding author.
